# Prognostic genes related to mitochondrial dynamics and mitophagy in diffuse large B-cell lymphoma are identified and validated using an integrated analysis of bulk and single-cell RNA sequencing

**DOI:** 10.3389/fimmu.2025.1686948

**Published:** 2025-10-09

**Authors:** Qingjiao Chen, Mingui Chen, Jizhen Wang, Jinfeng Dong, Apeng Yang, Xiaolin Zhu, Qiaoxian Lin, Jinlong Huang, Guilan Lai, Meihong Zheng, Zhiyong Zeng, Junmin Chen, Junfang Lin, Xiaoqiang Zheng

**Affiliations:** ^1^ Department of Hematology, The First Affiliated Hospital, Fujian Medical University, Fuzhou, Fujian, China; ^2^ Department of Hematology, National Regional Medical Center, Binhai Campus of the First Affiliated Hospital, Fujian Medical University, Fuzhou, Fujian, China; ^3^ Key Laboratory of Laboratory Medicine, Fuzhou, Fujian, China; ^4^ Hongshan Town Community Healthcare Service Center, Fuzhou, Fujian, China

**Keywords:** diffuse large B-cell lymphoma, mitochondrial dynamics, mitophagy, mitochondrial homeostasis signature, single-cell prognostic stratification

## Abstract

**Background:**

While the link between mitochondrial homeostasis, specifically dynamics and mitophagy, and the progression of diffuse large B-cell lymphoma (DLBCL) has been suggested, their prognostic significance and functional networks remain unclear. This study aimed to investigate the role of mitochondrial dynamics-related genes (MDRGs) in DLBCL patient outcomes.

**Methods:**

Candidate MDGRs were identified via Weighted Gene Co-expression Network Analysis (WGCNA) and differential expression analysis using public RNA-seq data. A prognostic signature was established via LASSO-Cox regression, followed by proportional hazards assumption validation. Functional pathways, regulatory networks (including miR-1252-5p/NEAT1), and a risk-scoring model were analyzed. Model assessment included nomograms, immune cell infiltration, m6A regulator, and pharmacogenomics. Single-cell mapping was employed to characterize B-cell differentiation and spatial gene expression. Finally, the findings were validated using RT-qPCR on clinical samples.

**Results:**

Six lysosomal-enriched genes (*TCF7*, *CEBPA*, *BBC3*, *GALR3*, *BMP8B*, and *BAALC*) were identified as independent prognostic indicators. A composite model integrating our risk score and clinical parameters showed superior predictive accuracy (AUC > 0.8). High-risk DLBCL was characterized by altered M0 macrophage infiltration, YTHDC1-mediated m6A dysregulation, and dihydrotestosterone sensitivity. Single-cell analysis revealed an association between stage-specific B-cell differentiation and gene expression gradients. RT-qPCR confirmed the upregulation of *CEBPA*, *BBC3*, *GALR3*, *BMP8B*, and *BAALC* in DLBCL clinical samples.

**Conclusion:**

*TCF7*, *CEBPA*, *BBC3*, *GALR3*, *BMP8B*, and *BAALC* were identified as novel lysosomal pathway-enriched prognostic genes in DLBCL. Our validated composite model demonstrated strong predictive power. These findings establish an association between high-risk disease and specific tumor microenvironment alterations (M0 macrophages), epitranscriptomic dysregulation (m6A), and therapeutic vulnerabilities, providing valuable insights for refining prognosis and advancing targeted therapies for DLBCL.

## 1 Introduction

Diffuse large B-cell lymphoma (DLBCL), the leading subtype of non-Hodgkin lymphoma, exhibits aggressive clinical behavior, considerable molecular heterogeneity, and swift progression (1, 2). It poses a major global health threat due to its ability to involve various organs, high recurrence rates, and therapeutic resistance in a subset of patients (3). Current prognostic models, such as the International Prognostic Index (IPI), rely heavily on clinical parameters but fail to fully capture the biological complexity of DLBCL. Although only 60-70% of patients achieve lasting remission with standard first-line therapy, outcomes vary significantly across molecular subtypes like germinal center B-cell (GCB) and activated B-cell (ABC), and approximately 30-40% develop refractory or relapsed disease highlighting the need for better treatment strategies (4). Furthermore, the existing biomarkers (such as mutations in *MYC*, *BCL2*, *MYD88*, *NOTCH2*, *CREBBP*, *KMT2D*, *TP53*, *CD58*, and *PIM1*) exhibit limited predictive value for treatment response or mechanisms of drug resistance (5–8). These limitations highlight critical gaps in our ability to provide personalized risk stratification and targeted therapies.

Identifying novel prognostic genes represents a pivotal step toward unraveling DLBCL pathogenesis, refining diagnostic precision, and advancing tailored therapeutic approaches. Emerging evidence suggests that dysregulated signaling pathways, epigenetic modifiers, and immune microenvironment interactions contribute to disease heterogeneity and therapeutic resistance (9). By integrating multi-omics data and functional validation (10, 11), the discovery of robust genetic signatures could guide the development of subtype-specific therapies, optimize treatment algorithms, and accelerate the translation of molecular insights into clinical applications (12). Such efforts promise to revolutionize DLBCL management and improve long-term survival in high-risk populations.

Mitochondria, often referred to as the cell’s powerhouses, are essential for energy production, metabolic regulation, and apoptosis (13). The balance between mitochondrial fusion and fission, which is tightly regulated, is crucial for maintaining mitochondrial quality, adapting to metabolic needs, and preserving cellular stability (14). Dysregulation of this dynamic equilibrium has been implicated in various pathologies, including neurodegenerative disorders, metabolic diseases, and cancer (15). Mitophagy, a selective form of autophagy responsible for eliminating dysfunctional mitochondria, exhibits dual regulatory effects in tumorigenesis (16). While this process suppresses tumor initiation through mitochondrial quality control (17), it paradoxically enhances cancer cell survival under stress conditions (such as hypoxia or chemotherapy) by maintaining metabolic plasticity and attenuating oxidative damage (18). This dual function underscores the context-dependent nature of mitophagy in cancer progression and therapy resistance.

Previous studies have found that m6A modification affects mitochondrial dynamics and function by regulating the translation of RNAs associated with mitochondrial function (19). In DLBCL, m6A modification regulates key target genes (such as *CHST11*) through *KIAA1429*, thereby influencing the Hippo-YAP signaling pathway, which modulates tumor cell proliferation, apoptosis, and disease progression (20). In conjunction with the role of Bcl-2 in DLBCL—where Bcl-2 maintains cell survival and prevents apoptosis by regulating mitochondrial outer membrane permeability and calcium ion homeostasis (21)—its overexpression enhances cancer cell survival. Thus, the interaction between m6A modification and mitochondrial dynamics may play an important role in the proliferation and survival of DLBCL, offering potential targets for future cancer therapies.

Emerging evidence suggests that mitochondrial dynamics and mitophagy are intricately linked to the pathogenesis of DLBCL (22). For instance, CD30, a transmembrane protein overexpressed in DLBCL, has been shown to activate BNIP3-mediated mitophagy, thereby shielding tumor cells from mitochondrial dysfunction and apoptosis induced by metabolic stress (23). Besides, imbalances in mitochondrial fission/fusion proteins (e.g., DRP1, MFN1/2) may contribute to chemoresistance by altering mitochondrial morphology and metabolic pathways critical for lymphoma cell survival (24). Studies have also highlighted the role of mitochondrial dynamics in modulating oxidative phosphorylation and glycolysis, processes that influence DLBCL aggressiveness and microenvironment interactions (25). Despite these advances, the precise molecular mechanisms by which mitochondrial dynamics-related genes (MDRGs) and mitophagy-related genes (MRGs) govern DLBCL progression remain poorly understood. Unraveling how mitochondrial dynamics and mitophagy intersect with DLBCL biology could reveal novel vulnerabilities, such as targeting mitochondrial plasticity to disrupt energy metabolism or enhance drug sensitivity. Such investigations hold transformative potential for overcoming therapeutic bottlenecks and improving patient outcomes in this heterogeneous malignancy.

Single-cell RNA sequencing (scRNA-seq) (26) is a transformative technology that facilitates high-resolution profiling of gene expression in individual cells, revealing transcriptional heterogeneity in complex tissues (27). By dissecting cellular diversity, scRNA-seq facilitates the reconstruction of cellular evolutionary trajectories during tumor development, identifying rare subpopulations (such as therapy-resistant clones), and mapping dynamic transitions between cell states (26). This approach also illuminates cell-cell communication networks by decoding ligand-receptor interactions and signaling crosstalk within the tumor microenvironment (28), thereby revealing mechanisms underlying immune evasion, stromal remodeling, and metastatic niches (29). In the context of DLBCL, scRNA-seq has uncovered novel subtypes (30), traced clonal evolution during relapse, and exposed microenvironmental reprogramming linked to immune checkpoint resistance (31), offering unprecedented insights into disease complexity and therapeutic vulnerabilities.

By integrating bulk and single-cell RNA sequencing data from public databases, this study systematically identified MDRGs and MRGs. A Cox prognostic model was built using the least absolute shrinkage and selection operator (LASSO) regression algorithm, which stratified patients according to survival outcomes and was subsequently validated for clinical relevance. Notably, this work is the first to establish a connection between two crucial biological processes—mitochondrial dynamics and mitophagy—and both prognosis and tumor microenvironment modulation in DLBCL, leading to the discovery and validation of novel prognostic biomarkers. Collectively, these findings not only deepen our insight into the role of mitochondrial plasticity in DLBCL pathogenesis but also offer a conceptual framework for advancing personalized immunotherapy and refining prognostic tools.

## 2 Materials and methods

### 2.1 Data acquisition

Among the datasets used in this study, GSE32018 (GPL6480) and GSE11318 (GPL570) datasets were classic bulk RNA sequencing (RNA-seq) datasets from the Gene Expression Omnibus (GEO) database (https://www.ncbi.nlm.nih.gov/geo/). The GSE32018 dataset contained 199 DLBCL tissue samples (DLBCL group) and 7 normal lymph-node tissue samples (control group) from individuals. Besides, the GSE32018 dataset contained 199 DLBCL tissue samples from individuals, which comprised clinical and survival information (the sample with a survival time of 0 was excluded). In addition, another RNA-seq dataset, the TCGA-DLBCL dataset, which included 47 DLBCL tissue samples from individuals (with all samples containing information on clinical outcomes and survival), was acquired from The Cancer Genome Atlas (TCGA) database (https://portal.gdc.cancer.gov/) (June 5, 2024). On the other hand, a dataset from single-cell RNA sequencing (scRNA-seq), which included 2 DLBCL tissue samples (DLBCL1 and DLBCL2) (DLBCL group) and 2 normal paraneoplastic tissue samples (rLN1 and rLN2) (control group) from individuals, was achieved from the heiDATA database (https://heidata.uni-heidelberg.de/) (**Supplementary Figure S1**).

Moreover, 23 mitochondrial dynamics-related genes (MDRGs) were obtained from the literature (32) (**Supplementary Table S1**). The integration of genes from the Reactome database (http://www.reactome.org), including mitophagy (R-HSA-5205647), pink1-prkn-mediated mitophagy (R-HSA-5205685), and receptor-mediated mitophagy (R-HSA-8934903), yielded 29 MRGs for downstream analysis (**Supplementary Table S2**).

### 2.2 Analysis of gene co-expression networks with weighted methods (WGCNA)

The GSE32018 dataset was analyzed using the ssGSEA algorithm from the GSVA package (v 1.46.0) (33) to calculate MDRGs and MRGs scores of all samples, aiming to assess the connection between mitochondrial dynamics, mitophagy and DLBCL progression. The Wilcoxon rank sum test was used to evaluate score differences between the DLBCL and control groups (*P*< 0.05). Furthermore, after removing unqualified samples by conducting hierarchical clustering analysis on all samples in the GSE32018 dataset via GoodSamplesGenes, WGCNA was executed with the WGCNA package (v 1.71) (34), yielding genes (among MDRGs and MRGs) associated with DLBCL. Specifically, to establish a scale-free co-expression gene network, optimal soft thresholding was then set according to the scale-free fit index (signed R2) and mean connectivity (close to 0) through the pickSoftThreshold function. Subsequently, genes were divided into different modules according to the hybrid dynamic tree cutting criteria, with a minimum number of 50 genes per gene module and a module fusion threshold of 0.25. Afterward, the MDRGs and MRGs were respectively used as phenotypes. The Spearman function was applied to assess correlations between module eigengene (ME) scores of modules and phenotypes, and genes within the module that had ME scores strongly correlated with both phenotypes were identified as potential key module genes(|correlation coefficients (cor)|> 0.8, *P*< 0.05). Following a comprehensive analysis of gene-module interactions and module-trait relationships, we identified critical module genes showing significant connections with MDRGs and MRGs based on predefined criteria (|module membership (MM)| > 0.8, and |gene significance (GS)| > 0.6).

### 2.3 Analyses of functional and protein-protein interaction

The limma package (v 3.54.0) (35) was applied to the GSE107943 dataset to perform differential expression analysis, aiming to identify differentially expressed genes (DEGs) between DLBCL and control groups (|log2fold change (FC)| > 2, and adjusted *P*< 0.05). Volcano plot visualization and hierarchical clustering analysis were implemented using ggplot2 ggplot2 (v 3.4.1) (36) and pheatmap (v 1.0.12) (37) packages, respectively, with the top 10 most significantly upregulated and downregulated DEGs selected based on |log2FC|. Afterward, gene screening was conducted using the ggvenn package (v 0.1.9) (38) to detect MDRGs-and MRGs-associated biomarkers in DLBCL. Candidate genes were defined as those demonstrating overlap between DEGs and core module genes through Venn analysis.

Subsequently, functional characterization of candidate genes was systematically performed through ontological enrichment analysis. Utilizing the clusterProfiler package (v 4.2.2) (39), we conducted Gene Ontology (GO) annotation encompassing biological processes (BP), cellular components (CC), and molecular function (MF) categories, along with Kyoto Encyclopedia of Genes and Genomes (KEGG) pathway analyses (*P*< 0.05). The enrichment landscape was visualized by GOplot (v 1.0.2) (40), depicting the five most enriched GO terms per category and the top twenty KEGG pathways.

To explore the interactions of the candidate genes at the protein level, the Search Tool for the Retrieval of Interacting Genes/Proteins (STRING) database (https://string-db.org/) was employed to establish a PPI network (confidence score > 0.4). The Cytoscape software (v 3.10.2) (41) was then used to visualize proteins with high-quality interactions.

### 2.4 Molecular regulatory network construction and gene set enrichment analysis for prognostic genes

To assess the potential value of MDRGs and MRGs for predicting overall survival (OS) of DLBCL patients, within the GSE11318 dataset, the survival package (v3.3.1) (42) was applied to conduct univariate Cox analysis on candidate genes (hazard ratio (HR) ≠1, *P*< 0.2). A threshold of *P*< 0.2 was adopted for variable screening to avoid overlooking potential confounding factors or variables with weak effects. This approach aligns with recommendations in statistical theory regarding variable selection (43, 44) and has been applied in practical research settings (45). The genes that remained were tested for the proportional hazards (PH) assumption (*P* > 0.05) via the coxph function. The results of univariate Cox analysis were visualized by the forest plot package (v3.1.1) (46), and the genes that met the PH assumption criteria were defined as candidate prognostic genes. Then, the glmnet package (v4.1.2) (46) was employed for a 10-fold cross-validation LASSO regression to analyze candidate prognostic genes. The remaining genes were subjected to backward elimination (HR ≠1, *P*< 0.1), identifying prognostic genes linked to mitochondrial dynamics and mitophagy.

Besides, GSEA was performed on each prognostic gene to understand their biological roles in DLBCL. Specifically, the psych package (v2.2.9) (47) facilitated a Spearman correlation analysis between prognostic genes and all the other genes, arranging them by |cor| in descending order. Subsequently, GSEA was performed using the clusterProfiler package (v4.2.2) (|normalized enrichment score| >1, *P*< 0.05). The gene set referenced was “c2. cp.kegg.v7.5.1. symbols.gmt”, sourced from the Molecular Signatures Database (MSigDB) (https://www.gsea-msigdb.org/gsea/msigdb). The leading five significant pathways of each prognostic gene were visualized by the enrichplot package (v1.18.3) (48), respectively. On the other hand, for an understanding of the molecular regulatory mechanisms of prognostic genes in DLBCL, miRDB (https://mirdb.org/) and miRanda (http://mirtoolsgallery.tech/mirtoolsgallery/node/1055) databases were applied to predict microRNAs (miRNAs) targeting prognostic genes, and the predicted miRNAs were then applied to predict corresponding long non-coding RNAs (lncRNAs) in the lncBase database (www.microrna.gr/LncBase). Besides, the NetworkAnalyst database (https://www.networkanalyst.ca/) was employed to forecast transcription factors (TFs) targeting prognostic genes. Finally, the lncRNA-miRNA-mRNA and TF-mRNA regulatory networks were visualized using Cytoscape software (v3.10.2).

### 2.5 Risk model establishment and verification

The risk model was formulated using the GSE11318 dataset and employed backward elimination to select prognostic genes related to mitochondrial dynamics and mitophagy. The risk score for each patient was calculated using the following formula:


$$\text{Riskscore }\left(\text{patients}\right)\;=\;\sum_{i=1}^{n}\;{\text{Expression}}_{\text{Genei}}\;\times \;{\text{Coefficient}}_{\text{Genei}}$$

Where, n represents the number of prognostic genes, and i denotes the serial number of each gene.

Based on an optimal threshold for these risk scores, DLBCL samples were stratified into high-risk (HRG) and low-risk (LRG) groups. The ggplot2 package (v3.4.1) was used to illustrate the distribution of risk scores and survival status in these two groups. Besides, expression trends of prognostic genes were also illustrated by ComplexHeatmap (v2.15.1) (37). Kaplan-Meier (KM) survival curves were generated for the two groups using the survival package (v 3.3.1), and survival differences were compared using a log-rank test (*P*< 0.05). Moreover, receiver operating characteristic (ROC) curves were plotted using the survivalROC package (v1.0.3) (49) for survival at 1, 3, and 5 years (an area under the curve (AUC) > 0.6 indicated good predictive performance). Besides, the TCGA-DLBCL dataset was used to validate the risk model, assessing its accuracy and generalizability.

### 2.6 Establishment of a nomogram and analysis of independent prognostic factors

In GSE11318 dataset, for screening independent predictors of prognosis and assessing the clinical use of risk assessment scores related to mitochondrial dynamics and mitophagy, age, gender, lactate dehydrogenase (LDH) ratio, extranodal, Eastern Cooperative Oncology Group performance status (ECOG PS), stage, and risk score were sequentially subjected to univariate Cox analysis (HR ≠1, *P*< 0.05), PH assumption test (*P* > 0.05), and multivariate Cox analysis (HR ≠1, *P*< 0.05) via survival package (v 3.3.1). The remaining factors were defined as independent prognostic factors and visualized by the forestplot package (v3.1.1). The rms package (v6.5.0) (50) was employed to establish a nomogram model that combines the risk score with clinical features derived from independent prognostic factors, aiming to predict 1, 3, and 5-year survival probabilities of DLBCL patients. Besides, the ROC curves (AUC > 0.6) were plotted using pROC (v1.18.0) (51) to verify model discrimination.

### 2.7 Immune cell infiltration, m6A-related, and drug sensitivity analyses

Using the GSE11318 dataset, the tumor microenvironment (TME) of HRG and LRG was explored. The CIBERSORT algorithm was used to evaluate the differences in the abundance of 22 types of immune cells (52) between HRG and LRG, excluding samples with *P* values greater than 0.05. Differential immune infiltrating cell types were then obtained through the Wilcoxon rank sum test (*P*< 0.05). Next, the psych package (v 2.2.9) was applied to conduct Spearman correlation analysis among differential immune cell types and prognostic genes (|cor| > 0.3, *P*< 0.05). Subsequently, expression differences in 20 m6A-related genes (53) (*VIRMA* was replaced with *KIAA1429*) between HRG and LRG were evaluated via the Wilcoxon rank sum test (*P*< 0.05). Similarly, the psych package (v 2.2.9) was employed to analyze the correlations among differentially expressed m6A-related genes, and prognostic genes were analyzed via Spearman correlation analysis (|cor| > 0.3, *P*< 0.05). All results were visualized using the ggplot2 package (v 3.4.1).

Finally, the oncoPredict package (v0.2) (54) was applied to calculate the 50% inhibitory concentration (IC_50_) of 198 chemotherapy drugs from the Genomics of Drug Sensitivity in Cancer (GDSC) database (https://www.cancerrxgene.org) for DLBCL patients in HRG and LRG groups. In addition, drugs with notable differences in IC_50_ between HRG and LRG were acquired using the Wilcoxon rank sum test (*P*< 0.05). The top 20 medications with significant changes in IC_50_ between groups were visualized. Besides, the psych package (v 2.2.9) was utilized to conduct Spearman correlation analysis (|cor| > 0.3, *P*< 0.05) among differential drugs and prognostic genes. These results were visualized by the ggplot2 package (v 3.4.1).

### 2.8 The scRNA-seq data processing, intercellular interaction analysis, and cell trajectory analysis

Further investigations were performed on the scRNA-seq dataset to explore the expression of prognostic genes linked to mitochondrial dynamics and mitophagy at the single-cell level.

First, the PercentageFeatureSet function in the Seurat package (v5.0.1) (55) was used to filter scRNA-seq data (5%< nFeature RNA percent of mitochondrial genes< 5%). Specifically, data evaluation and cell screening were conducted based on the following 3 parameters: nFeature_RNA (the number of genes detected in each cell, with the lowest and highest 5% of all its values used to identify cells with low expression levels or poor quality, and those with high expression levels or other issues respectively), nCount_RNA (the total number of unique molecular identifiers (UMIs) in each cell, and the highest 5% of its values were used to identify cells with abnormally high UMI counts due to high expression or technical problems), and the proportion of mitochondrial genes (with a cutoff of less than 0.05 set to screen for normal cells). The quality control chart was generated by the ggplot2 package (v3.4.1). Next, the PercentageFeatureSet function in the Seurat package (v5.0.1) was applied to identify 2,000 genes with the largest variation (the 10 genes with the largest variation were labeled in a volcano plot generated by the LabelPoints function). Furthermore, the ScaleData function in the Seurat package (v5.0.1) was used to normalize the samples. Subsequently, an analysis using principal component analysis (PCA) was conducted on the 2000 most highly variable genes. The dimensionality reduction results were visualized in an inflection point plot via the Elbowplot function. Next, the PCA replacement test was conducted through the Jackstraw function and the principal components (cells) that could be used for later analyses (*P*< 0.05).

After PCA downscaling, the uniform manifold approximation and projection (UMAP) clustering method was used to identify cell clusters (resolution =0.1). Afterwards, cell annotation was conducted. Specifically, the marker genes from the CellMarker database (http://bio-bigdata.hrbmu.edu.cn/CellMarker) were applied to annotate the cell types of the different clusters. The annotation results were visualized in the UMAP plot, and the expression of marker genes in different cell types was illustrated via the ggplot2 package (v3.4.1). Subsequently, the R package CellChat (v 1.6.1) (56) was used to analyze ligand-receptor pairs and molecular interactions among different annotated cell types in the disease and control groups (*P*< 0.05, Log2 (mean (Molecule1, Molecule2)) ≥ 0.1), and visualized the communication networks to compare the number and strength of communication networks between the two groups.

Based on all samples, the distribution of prognostic genes was analyzed, and the data were presented in UMAP plots. Besides, the expression differences of prognostic genes between DLBCL and control groups were compared by employing the Wilcoxon rank sum test (*P*< 0.05). Notably, the cell type exhibiting significant differences in the expression of most prognostic genes between groups was defined as the key cell type. Furthermore, to reveal the related biological functions of key cell types, functional analysis was conducted via the ReactomeGSA package (v1.16.1) (57) (*P*< 0.05).

Based on all samples, the key cell type was further clustered into distinct subtypes by UMAP downscaling. Subsequently, the Monocle2 package (v2.26.0) (58) was employed to conduct cell trajectory analysis, by which the differentiation of key cell types was simulated, and the expression trends of prognostic genes at different developmental stages in key cell types were illustrated.

### 2.9 Annotation of key cell subpopulations and pseudo-time analysis

To investigate gene expression changes in each cell during critical cellular state transitions, cell trajectory and pseudotime analysis were performed using the plot_genes_in_pseudotime algorithm from the R package monole (v 2.26.0) (59). To reduce dimensionality, the RunPCA function from the R package Seurat (v 5.0.1) (55) to perform PCA analysis on the selected highly variable genes. The Jackstraw function was used for significance testing (*P*< 0.05), and the ElbowPlot function was employed to rank principal components (PCs), selecting effective PCs for subsequent analysis.

To understand key cell heterogeneity, the FindClusters function in the R package Seurat (v 5.0.1) was used to perform cluster analysis on the PCs (resolution set to 0.2), yielding distinct subpopulations. The RunUMAP function visualized the clustering results. Subsequently, key cell subpopulations were annotated based on marker genes provided by the FindAllMarkers function, referencing literature (60). After removing non-key cell types, an UMAP projection plot was generated. Subsequently, pseudotime trajectory analysis was performed on key cell subpopulations, and developmental trajectory plots were visualized with color coding based on pseudotime, differentiation stage, and developmental time. Finally, the plot_genes_in_pseudotime function from the monole package (v 2.26.0) was used to plot the dynamic trends of prognostic gene contributions during cell differentiation.

Additionally, metabolic enrichment analysis was performed to explore the metabolic characteristics of different key cell subpopulations.

### 2.10 The process of reverse transcription-quantitative PCR

To validate the levels of expression for prognostic genes in clinical samples, RNAs of 5 DLBCL tissue samples and 5 control samples from individuals were isolated using TRIzol reagent (R401-01, Ambion, America). The collection was performed at the First Affiliated Hospital of Fujian Medical University in Fujian province. The isolated RNAs were then used for cDNA synthesis employing the Hifair^®^ III 1st Strand cDNA Synthesis SuperMix for qPCR kit (11141ES60, Yisheng, China). Subsequently, RT-qPCR was executed using 2×Universal Blue SYBR Green qPCR Master Mix (G3326-05, Servicebio, China). Primers for prognostic genes and the internal reference gene (GAPDH) were listed in **Supplementary Table S3**. The reaction system and program were performed according to the reagent’s instructions. Following the RT-qPCR procedure, the 2^-ΔΔCт^ method was applied to determine relative expression levels. A t-test (*P*< 0.05) was applied to evaluate differences between groups, and data visualization was conducted using Graphpad Prism 5 software (v8.0) (61). Ethical approval for this study was granted by the Branch for Medical Research and Clinical Technology Application, Ethics Committee of the First Affiliated Hospital of Fujian Medical University. Approval No. MRCTA, ECFAH of FMU [2023]350. Written informed consent was obtained from all participants.

### 2.11 Statistical analysis

R language (v 4.3.1) was utilized to perform all bioinformatic analyses. Besides, the Wilcoxon rank sum test, the log-rank test, and the t-test were employed in this study to assess differences between groups, setting the significance threshold at *P*< 0.05. To validate the appropriateness of the Wilcoxon rank sum test, the data was examined for normality using the Shapiro-Wilk test and a QQ plot. Results indicated that the data did not follow a normal distribution (W = 0.9046, *P*< 2.2e-16, **Supplementary Figure S2**).

## 3 Results

### 3.1 Candidate genes in DLBCL related to mitochondrial dynamics and mitophagy and their associated functions

Based on the GSE32018 dataset, the DLBCL group exhibited significantly higher scores for MDRGs and MRGs compared to the control group, which revealed that mitochondrial dynamics and mitophagy were strongly associated with DLBCL progression (**Supplementary Figures S3A, B**). Furthermore, after removing unqualified samples by sample clustering (cutHeight = 290) (**Supplementary Figure S3C**), WGCNA was undertaken to identify key module genes associated with mitochondrial dynamics and mitophagy. The optimal soft thresholding was set at 8 according to the scale-free fit index (R^2^ = 0.9) and mean connectivity (close to 0). A gene co-expression network was established with 14 gene modules, excluding the gray module (**Supplementary Figures S3D, E**). Notably, candidate key module genes strongly correlated with MDRGs and MRGs were obtained from blue (cor (MDRGs) = 0.84, cor (MRGs) = 0.92) and brown (cor (MDRGs) = -0.91, cor (MRGs) = -0.82) modules (*P*< 0.0001) (**Supplementary Figures S3F, G**). Subsequently, 931 key module genes were selected based on the predefined criteria(|MM| > 0.8, |GS| > 0.6) (**Supplementary Figures S3H, I**).

Besides, analysis of the GSE32018 dataset identified 158 DEGs in the DLBCL group, including 14 up-regulated and 144 down-regulated genes (*P*< 0.05) (**Supplementary Figure S4A**). Afterward, 98 candidate genes were identified from the intersection of DEGs and key module genes (**Supplementary Figure S4B**). Importantly, candidate genes were significantly enriched in GO entries and KEGG pathways, including cytokine-mediated signaling pathway and lymphocyte differentiation (*P*< 0.05) (**Supplementary Figure S4C**). Furthermore, the PPI network revealed 42 proteins formed 41 interacting pairs, including proteins such as COX6A2 and UTS2R (**Supplementary Figure S4D**). These findings provide a better understanding of the multiple roles of mitochondrial dynamics and mitophagy in DLBCL progression.

### 3.2 Crucial functional pathway and elaborate molecular regulatory networks of six prognostic genes in DLBCL

**Figure 1:**
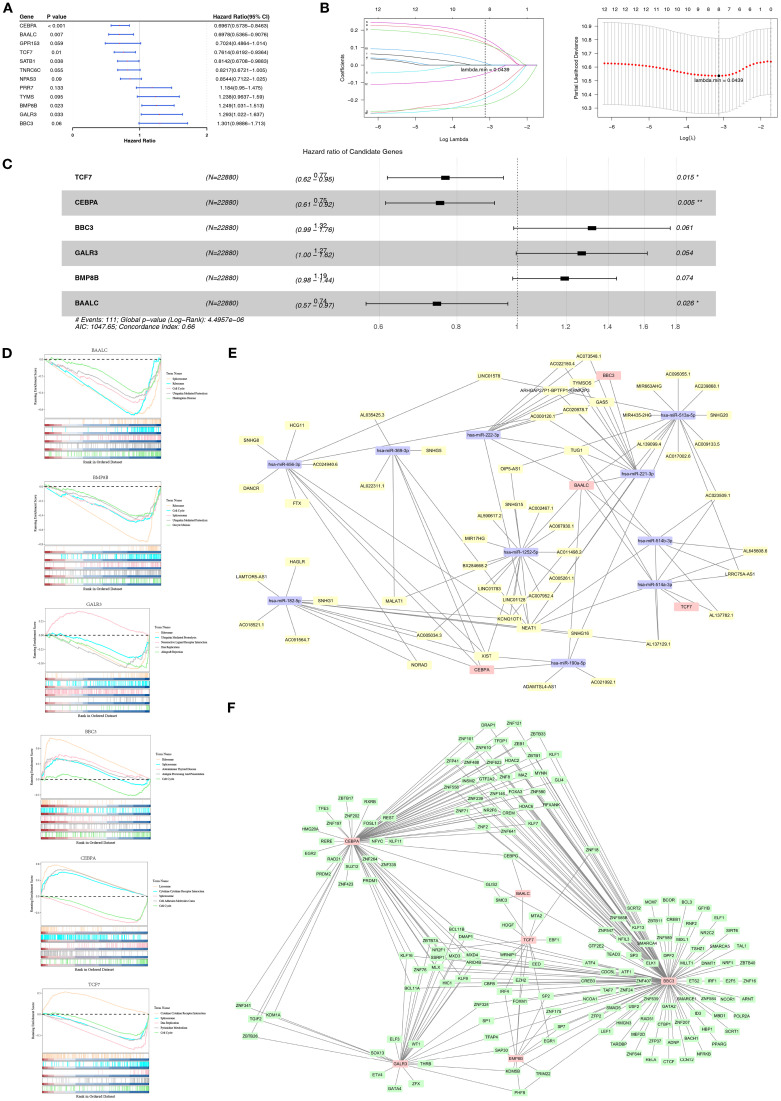
Prognostic gene screening and regulatory network in DLBCL. **(A)** Forest plot of the 12 significant module genes identified using Univariate Cox analysis (*P*< 0.2) and PH assumption test (P > 0.05) in DLBCL patients. **(B)** LASSO regression retained eight candidate genes, including *TCF7*, *CEBPA*, *BBC3*, *GALR3*, *BMP8B*, *PRR7*, *BAALC*, and *NPAS3*. **(C)** Backward elimination (*P*< 0.1) selected six prognostic genes linked to MDRGs, including *TCF7*, *CEBPA*, *BBC3*, *GALR3*, *BMP8B*, and *BAALC*. **(D)** GSEA revealed significant enrichment of prognostic genes in pathways, indicating that mitochondrial dynamics and mitophagy modulate DLBCL via these pathways. **(E)** An lncRNA-miRNA-mRNA network was constructed. **(F)** A TF-mRNA network showed that prognostic genes were regulated by transcription factors. “*” indicates P < 0.05, and “**” indicates P < 0.01.

Based on the GSE11318 dataset, 12 candidate prognostic genes associated with OS of DLBCL patients were identified through univariate Cox analysis (*P*< 0.2) and PH assumption test (*P* > 0.05) (**Figure 1A**, **Supplementary Table S4**). After LASSO regression analysis, 8 candidate prognostic genes (*TCF7, CEBPA, BBC3, GALR3, BMP8B, PRR7, BAALC*, and *NPAS3*) were retained (lambda.min = 0.0439) (**Figure 1B**). Following this, six prognostic genes linked to mitochondrial dynamics and mitophagy were identified using backward elimination (*P*< 0.1), including *TCF7, CEBPA, BBC3, GALR3, BMP8B*, and *BAALC (* **Figure 1C** *). TCF7, CEBPA*, and *BAALC* were associated with better prognosis (HR< 1), suggesting that they might inhibit DLBCL progression. Conversely, *BBC3*, *GALR3*, and *BMP8B* were associated with adverse prognosis (HR > 1), indicating they could facilitate the advancement of DLBCL.

Furthermore, biological pathways related to prognostic genes in DLBCL were discovered using GSEA. Specifically, prognostic genes were found to be associated with pathways like cytokine-cytokine receptor interaction, lysosome, ribosome, and spliceosome (**Figure 1D**). Based on the above findings, it can be inferred that mitochondrial dynamics and mitophagy exhibit pivotal roles in DLBCL progression by affecting these pathways.

Furthermore, regulatory elements targeting prognostic genes were predicted. The constructed lncRNA-miRNA-mRNA network comprised miRNAs (such as has-miR-1252-5p and has-miR-222-3p) targeting specific prognostic genes (*TCF7*/*CEBPA*/*BBC3*/*BAALC*) and lncRNAs (such as *NEAT1* and *XIST*) targeting specific miRNAs (**Figure 1E**). Besides, the TF-mRNA network revealed prognostic genes (*TCF7/CEBPA/BBC3/GALR3/BMP8B/BAALC*) that were regulated by specific TFs like *SMC3* and *FOXM1* (**Figure 1F**). These findings are crucial for elucidating the pathophysiological mechanisms related to mitochondrial dynamics and mitophagy in DLBCL.

### 3.3 Strong predictive power of MDRGs and MRGs for DLBCL prognosis demonstrated by a risk model

**Figure 2:**
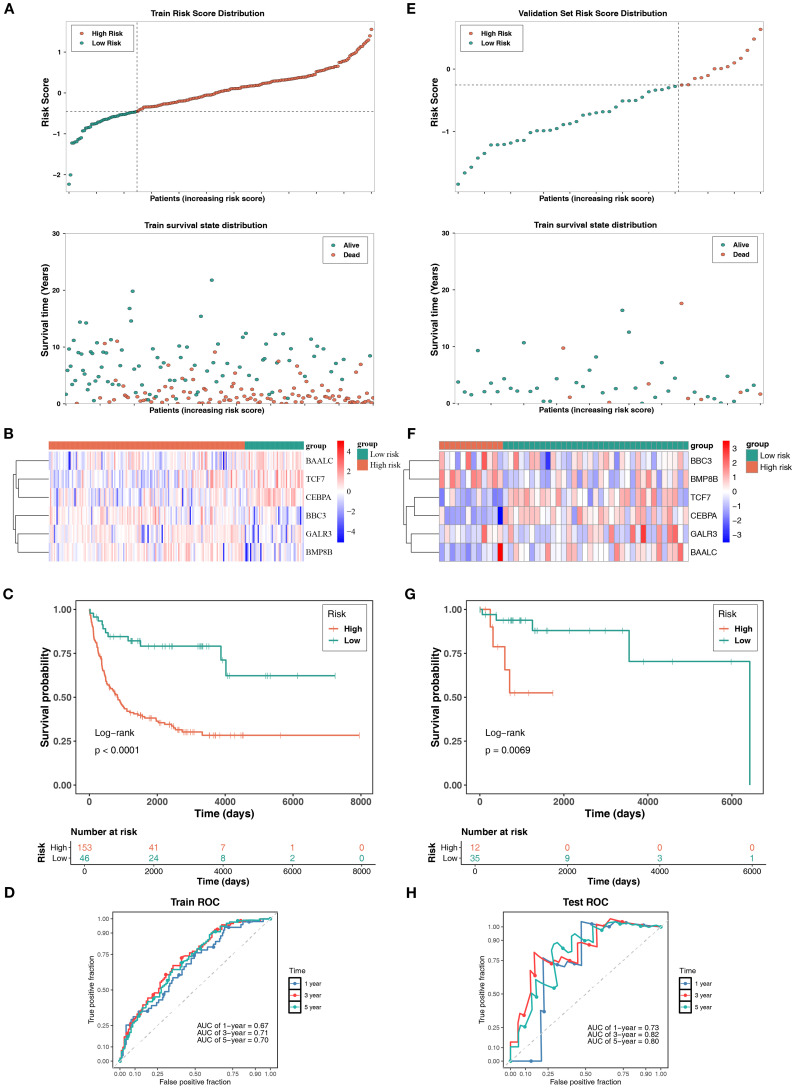
Risk model construction and validation model in DLBCL. **(A)** Risk score distribution and survival status indicated increased mortality with higher risk scores in DLBCL patients. **(B)** Prognostic gene expressions in different groups, with *BBC3*, *GALR3*, and *BMP8B* upregulated in HRG, and *BAALC*, *TCF7*, and *CEBPA* upregulated in LRG. **(C)** KM survival curves demonstrated significantly higher survival probability in LRG (*P*< 0.0001). **(D)** ROC curves showed AUCs > 0.6 at 1-, 3-, and 5-year intervals, confirming model efficacy. **(E-H)** Validation of the risk model was performed in the TCGA-DLBCL dataset.

Within the GSE11318 dataset, after obtaining prognostic genes, a risk model related to mitochondrial dynamics and mitophagy was constructed: risk score = (-0.26622943) × *TCF7* expression level + (-0.285915897) × *CEBPA* expression level + (0.276313141) × *BBC3* expression level + (0.238247261) × GALR3 expression level + (0.175169469) × *BMP8B* expression level + (-0.297403233) × *BAALC* expression level. Next, DLBCL patients were divided into HRG and LRG (153: 46) based on an optimal cutoff value of -0.4496605. The distribution of risk scores and survival status within risk groups illustrated that mortalities of DLBCL patients increased with increasing risk scores (**Figure 2A**). Notably, *BBC3*, *GALR3*, and *BMP8B* exhibited higher expression in HRG, while *BAALC*, *TCF7*, and *CEBPA* exhibited higher expression in LRG (**Figure 2B**). Besides, KM survival curves revealed that DLBCL patients in LRG exhibited markedly higher survival probabilities (*P*< 0.0001) (**Figure 2C**). Besides, the AUC of ROC curves at 1, 3, and 5 years all exceeded 0.6, reflecting the good prognostic performance of this risk model (**Figure 2D**).

Besides, the risk model underwent validation using the TCGA-DLBCL dataset. DLBCL patients in this dataset were classified into HRG and LRG (12: 35) using an optimal risk score threshold of -0.2549045. The results, including the risk score, survival status (**Figure 2E**), prognostic gene expression trends (except *GALR3*) (**Figure 2F**), KM survival curves (*P*< 0.01) (**Figure 2G**), and ROC curves (AUC all exceeded 0.7) (**Figure 2H**), were largely aligned with the GSE11318 dataset. The risk model related to mitochondrial dynamics and mitophagy demonstrated robust generalizability, suggesting its huge potential for tailored prognostic evaluation in the clinical management of DLBCL.

### 3.4 Establishment of a nomogram integrating risk scores and clinical characteristics for accurate prediction

**Figure 3:**
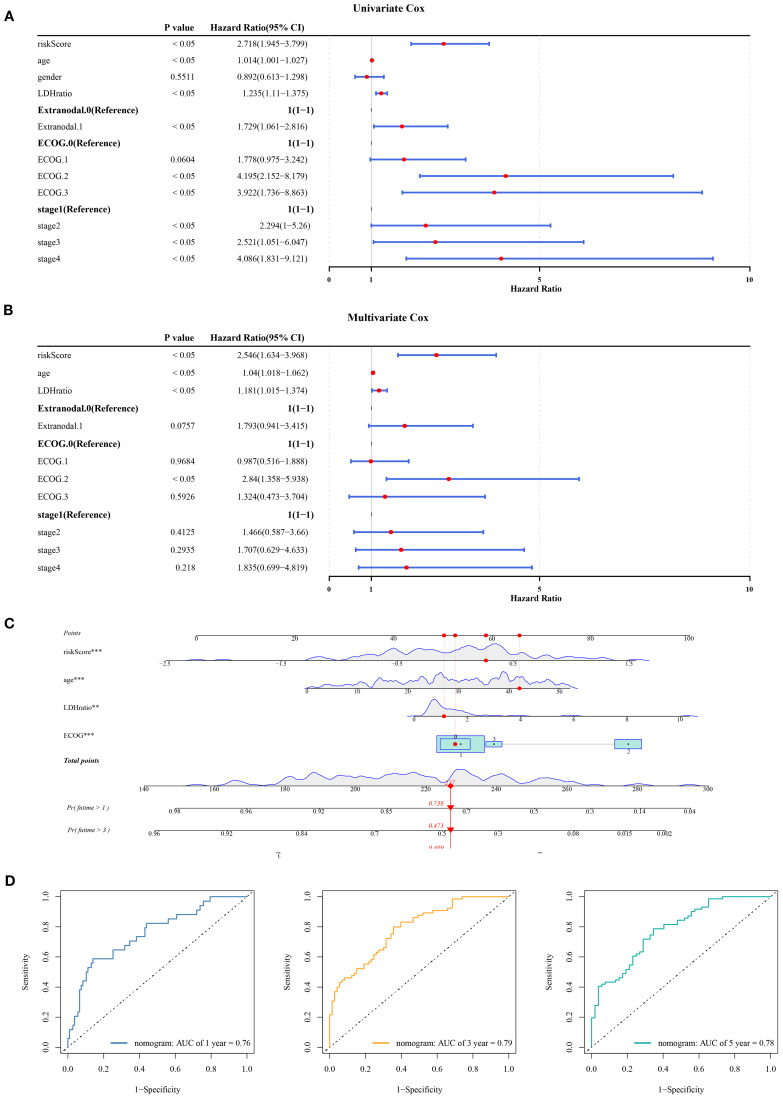
Nomogram and independent prognostic analysis in DLBCL. **(A)** Univariate Cox analysis identified 6 prognostic factors, including age, LDH ratio, extranodal involvement, ECOG PS, stage, and risk score (*P*< 0.05). **(B)** Multivariate Cox analysis confirmed age, LDH ratio, ECOG PS, and risk score as independent prognostic factors (*P*< 0.05), all associated with adverse outcomes (HR > 1). **(C)** A nomogram integrating independent factors. **(D)** ROC analysis at 1-, 3-, and 5-year intervals. “**” indicates P < 0.01, and “***” indicates P < 0.001.

In the GSE11318 dataset, the clinical applicability of risk scores related to mitochondrial dynamics and mitophagy was evaluated by Cox regression analysis. Specifically, a total of 6 factors (age, LDH ratio, extranodal, ECOG PS, stage, and risk score) were identified as significant predictors of prognosis (*P*< 0.05) and satisfied the proportional hazards assumption (*P* > 0.05) (**Figure 3A**, **Supplementary Table S5**). Following this, these factors were evaluated through multivariate Cox analysis, with age, LDH ratio, ECOG PS, and the risk score identified as independent predictors of prognostic (*P*< 0.05) (**Figure 3B**). Notably, these factors were associated with an adverse prognosis of DLBCL patients (HR > 1). Subsequently, a nomogram integrating independent prognostic factors was established (**Figure 3C**). Notably, DLBCL patients with higher total points have lower chances of survival at 1, 3, and 5 years. ROC analysis showed that the AUC at 1, 3, and 5 years all exceeded 0.7 (**Figure 3D**), demonstrating that the discrimination ability of this nomogram model was superior, further highlighting the remarkable clinical utility of risk scores related to mitochondrial dynamics and mitophagy in prognostic evaluation of DLBCL.

### 3.5 Differential TME, expression of m6A-related genes, and drug sensitivities altered by risk scores

On the one hand, the TME profiles of HRG and LRG in the GSE11318 dataset were analyzed (**Supplementary Figure S5A**). Notably, 8 differential immune infiltrating cell types, including M0 macrophages and T follicular helper cells, were identified (*P*< 0.05) (**Supplementary Figure S5B**). Moreover, a strong correlation was observed among these differential immune infiltrating cell types. For example, the most significant positive correlation was observed between T follicular helper cells and regulatory T cells (Tregs)/gamma delta T cells (cor = 0.35). In contrast, the most significant negative correlation was observed between memory B cells and gamma delta T cells (cor = -0.45) (*P*< 0.01). Besides, the prognostic genes were markedly correlated with specific differential immune infiltrating cell types. For instance, *CEBPA* showed the strongest positive correlation with M0 macrophages (cor = 0.52), while the strongest negative correlation was observed between *CEBPA* and memory B cells (cor = -0.49) (*P*< 0.001) (**Supplementary Figure S5C**). The above findings suggest that the TME profiles of HRG and LRG are different and associated with the expression of prognostic genes and specific immune cells. Consequently, the prognostic genes related to mitochondrial dynamics and mitophagy could serve as potential new targets for personalized therapeutic approaches in DLBCL.

On the other hand, the expression of 10 m6A-related genes like *YTHDC1* and *HNRNPC* demonstrated significant differences between HRG and LRG (*P*< 0.05) (**Supplementary Figure S5D**) (*RBMX* was not expressed in the samples). Besides, the prognostic genes were notably associated with specific m6A-related genes. For instance, *BBC3* exhibited a significant negative correlation with *YTHDF3* (cor = -0.42), and *CEBPA* showed a significant negative correlation with *HNRNPA2B1* (cor = -0.40) (*P*< 0.001) (**Supplementary Figure S5E**). These results suggest that the prognostic genes could regulate tumor cell behavior by influencing the dynamic regulation of m6A modification, which in turn alters RNA stability, translation efficiency, or splicing patterns.

Regarding therapeutic drugs, the IC_50_ values for specific therapeutic drugs varied significantly between HRG and LRG (*P*< 0.05). The IC_50_ of drugs like dihydrorotenone, elephantin, and fulvestrant were significantly lower in LRG (*P*< 0.0001) (**Supplementary Figure S5F**). Notably, a lower IC_50_ value indicated greater drug efficacy. Notably, *CEBPA* exhibited markedly strongly positive correlations with most drugs, especially Vorinostat_1012 (cor = 0.56) (*P*< 0.001) (**Supplementary Figure S5G**). Consequently, these differential drug sensitivities might be due to different drug metabolism mechanisms affected by specific prognostic genes in DLBCL patients from varying risk groups.

### 3.6 Identification of key cell type (B cells) and relevant functions

**Figure 4:**
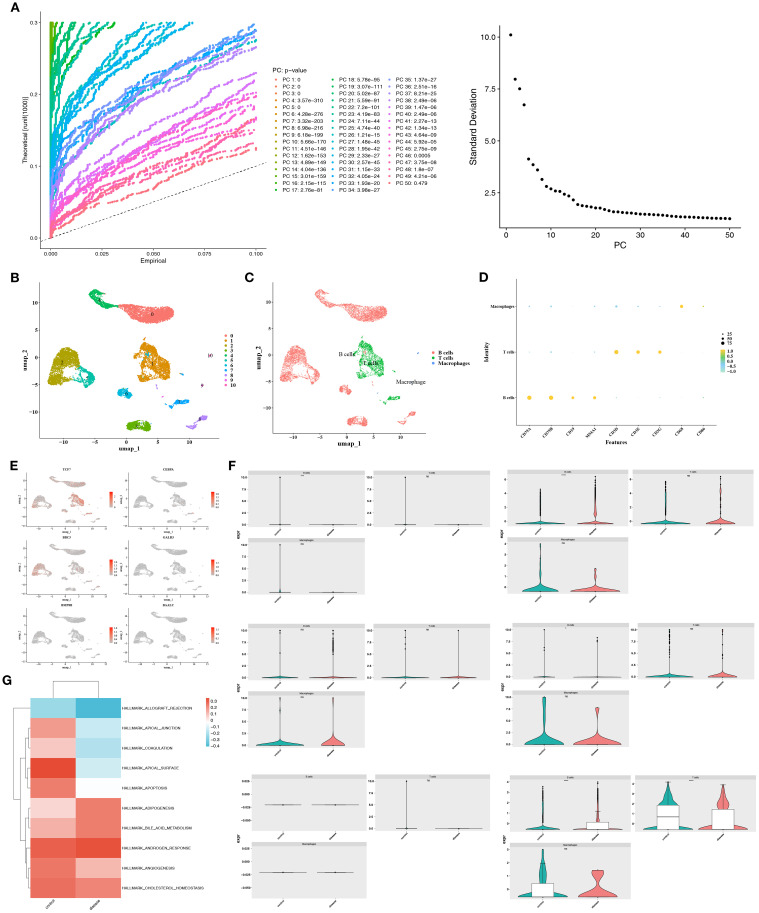
Single-cell annotation and key cell identification in DLBCL. **(A)** Top 20 principal components retained based on the elbow plot and PCA permutation test. **(B)** Principal component clustering identified 11 cell subpopulations. **(C)** Cell type annotation using marker genes. **(D)** Expression patterns of marker genes across annotated cell types. **(E)** Differential distribution of prognostic genes *TCF7* and *BBC3* in cell types. **(F)** B cells were defined as the key population due to significant inter-group differences in prognostic gene expression (*BAALC*, *BBC3*, *CEBPA*, *TCF7*). **(G)** Functional enrichment of B cells in pathways like allograft rejection, apical junction, and coagulation.

To investigate the mechanisms related to mitochondrial dynamics and mitophagy in DLBCL at a single-cell resolution, scRNA-seq data were filtered. The integrated data contained 12,087 cells and 23,432 genes (**Supplementary Figure S6**). The variability among these genes was assessed (**Supplementary Figure S7**), and the top 10 most variable genes were identified. PCA was performed for dimensionality reduction (**Supplementary Figure S8**). Both the inflection point plot and PCA replacement test indicated that the top 20 principal components should be retained for downstream analysis (**Figure 4A**). The retained principal components (cells) were subsequently clustered and labeled. These cells were classified into 11 clusters (**Figure 4B**). Based on the application of marker genes, cell clusters were further annotated into 3 types, including B cells (marker genes: *CD79A, CD79B, CD19, and MS4A1*), T cells (marker genes: *CD3D*, *CD3E*, and *CD3G*), and macrophages (marker genes: *CD68* and *CD86*) (**Figure 4C**). Besides, the expression of marker genes in different cell types was illustrated (**Figure 4D**). Furthermore, the distribution of two prognostic genes, *TCF7* and *BBC3*, was also mapped across the annotated cell types (**Figure 4E**). Notably, given that the B cells exhibited significant differences in the expression of most prognostic genes (*BAALC, BBC3, CEBPA, TCF7*) between the two groups, they were defined as the key cell type (**Figure 4F**). Moreover, it was found that B cells were notably associated with functions, encompassing allograft rejection, apical junction, and coagulation (**Figure 4G**). Cell communication analysis revealed that in the disease group, B cells exhibited a higher number of communication events with T cells, albeit with weaker intensity, while communication between B cells and themselves was stronger (**Supplementary Figure S9A**). Conversely, in the control group, communication between T cells and B cells appeared to be stronger. The absence of normal T-B cell communication in the disease group suggested that the disease might have induced T cell immune imbalance (**Supplementary Figure S9B**). Ligand-receptor interaction bubble plots further revealed that in the disease group, B cells most frequently communicated with themselves via the MIF pathway (**Supplementary Figure S9C**). In the control group, T-cell-B-cell communication also primarily occurred through the MIF pathway, exhibiting the highest communication probability (**Supplementary Figure S9D**).

### 3.7 Trajectories of B cells and the expression patterns of prognostic genes

**Figure 5:**
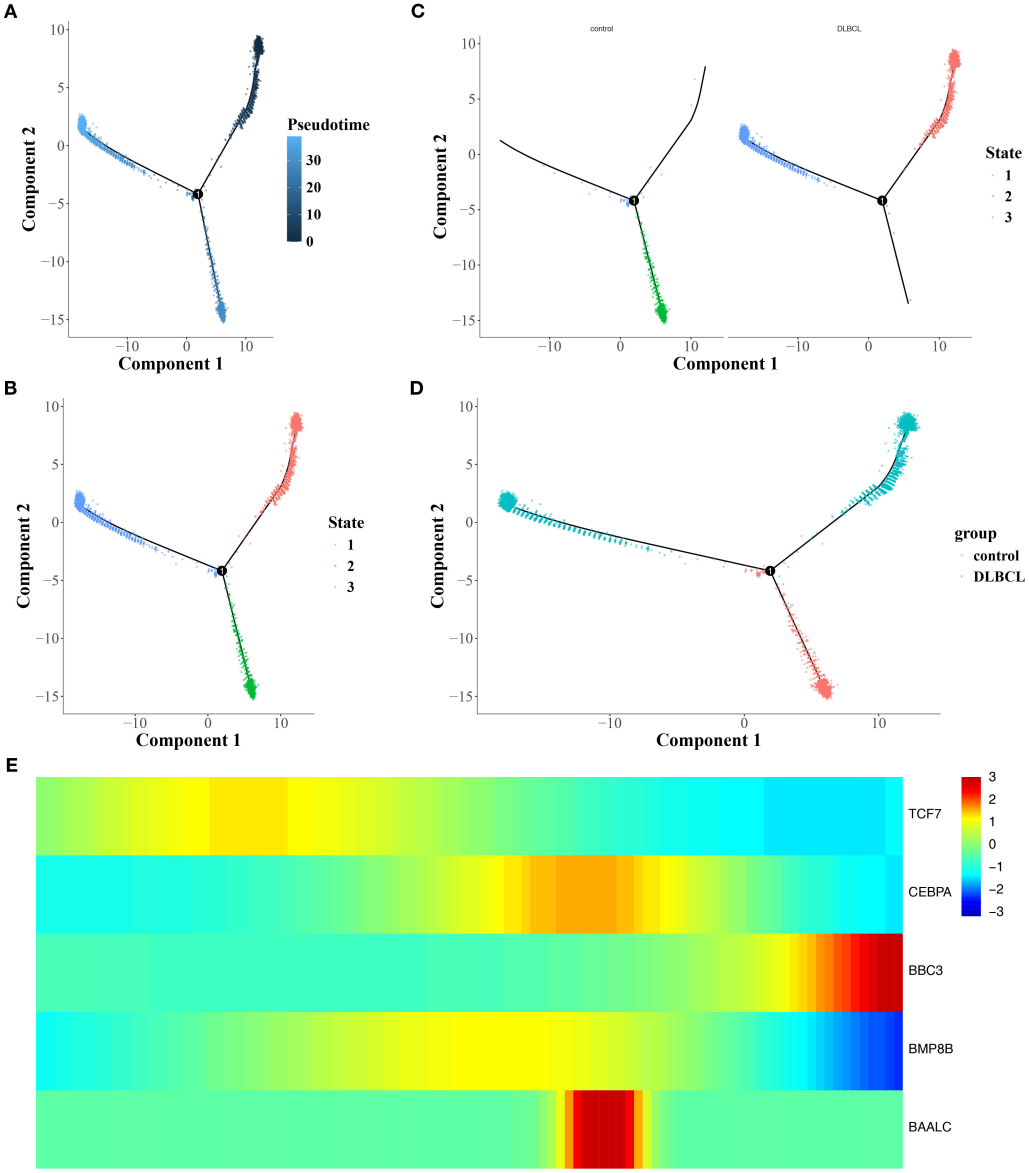
Trajectory analysis of B-cell differentiation and gene expression dynamics. **(A)** Pseudotime trajectory of B-cell differentiation, with darker hues indicating earlier differentiation stages. **(B, C)** Timeline diagram depicting the differentiation and development stages of different groups. **(D)** Differentiation and development trajectories of different groups. **(E)** Heat map of the interactions among different stages.

An analysis of B cell trajectories was performed, revealing their differentiation over time. The differentiation trajectory is visualized with a color gradient, where darker colors indicate earlier differentiation stages (**Figure 5A**). The analysis clearly revealed three distinct states of B cell differentiation (**Figures 5B, C**). In the differentiation trajectory, B cells from control samples predominantly clustered in the middle stage of differentiation, whereas those from DLBCL samples formed large aggregates in the early and late stages (**Figure 5D**).

Besides, it was found that *TCF7* and *BBC3* were highly expressed during the early and late stages of B cell differentiation, respectively. In addition, *CEBPA*, *BAALC*, and *BMP8B* showed elevated expression during the middle stage of B cell differentiation (**Figure 5E**). In summary, the influence of mitochondrial dynamics and mitophagy on DLBCL progression might be linked to the differentiation stages of B cells and the expression of prognostic genes across these stages.

### 3.8 Key cell types (B cells) annotated seven cell subpopulations

To explore prognostic gene expression changes within B-cell subpopulations, PCA analysis was first performed, selecting the top 10 PCs for subsequent analysis (**Supplementary Figures S10A, B**). Clustering analysis ultimately identified seven distinct cell subpopulations (**Supplementary Figure S10C**). Subsequently, annotation of these cell subpopulations yielded seven distinct cell types: IgE-MemB/PB, GC-LZ-like (SOX4+), GC-DZ (Cycling), FolB IgM+CD23+, Non-B (Epithelial-like), Non-B (Stromal/Metab.), and T (**Supplementary Figure S10D**, **Supplementary Table S6**). After removing contaminated cell types, four cell types were ultimately retained: FolB IgM+CD23+, GC-LZ-like (SOX4+), GC-DZ (Cycling), and IgE-MemB/PB (**Supplementary Figure S10E**).

Further analysis revealed that key cell differentiation trajectories originate from dark regions, with light regions representing cell types at the differentiation terminus. During differentiation, GC-DZ (Cycling) cells were present in both early and late stages. IgE-MemB/PB cells predominantly occupied the early stage, GC-LZ-like (SOX4+) cells primarily occupied the late stage, while FolB IgM+CD23+ cells resided in a lower branch. Furthermore, key cells were categorized into three stages: most cells resided in the red Stage 1, followed by green Stage 2, with a minority in blue Stage 3. Comparing cell distributions between disease and control groups revealed significantly more cells in the disease group, while the lower branches exhibited richer distribution in the control group (**Supplementary Figure S10F**).

**Figure 6:**
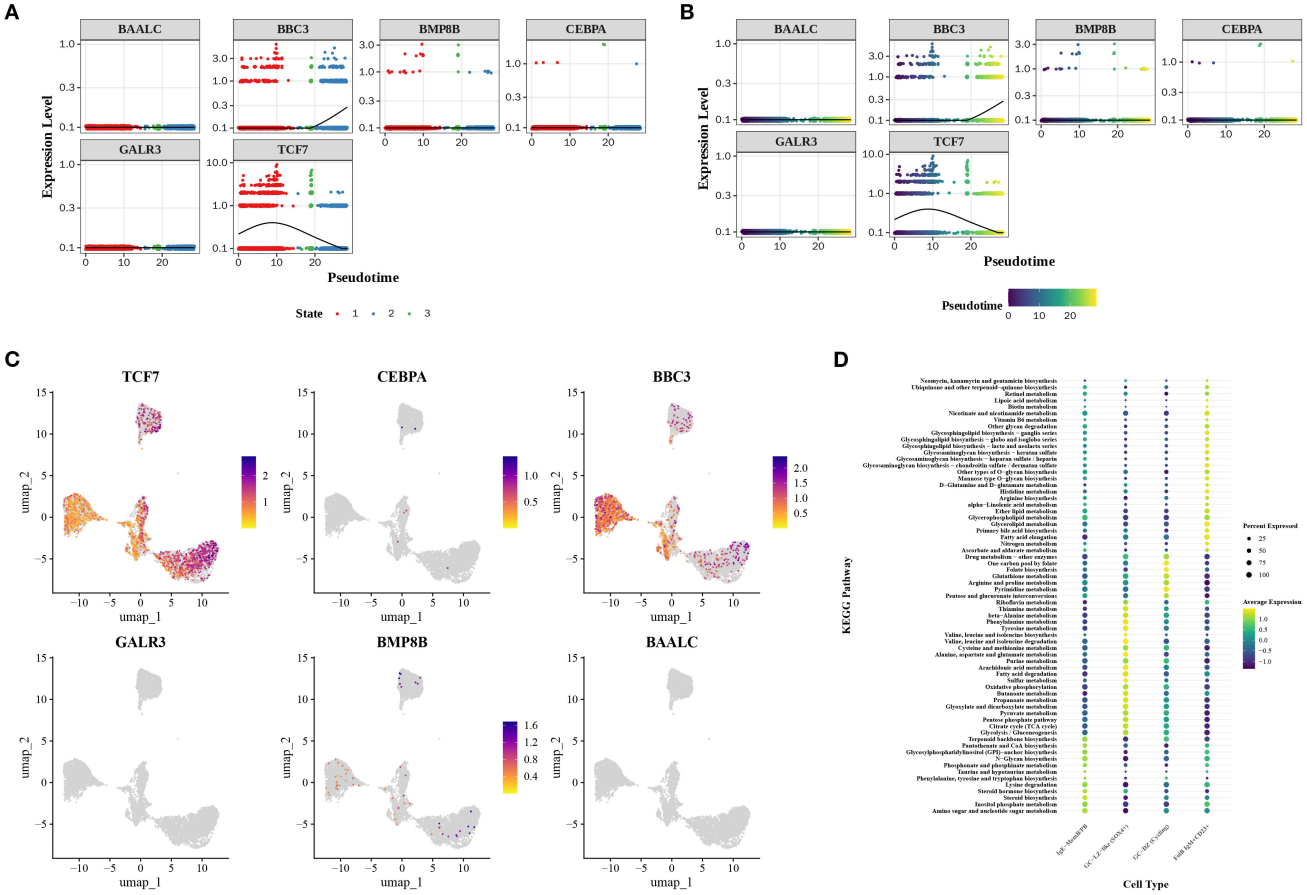
Prognostic gene expression and metabolic features in cellular subpopulations. **(A, B)** Expression heatmaps reveal dynamic changes in prognostic genes during cellular differentiation. **(C)** Expression distribution of prognostic genes in B cells. **(D)** Metabolic enrichment analysis of cellular subpopulations.

Regarding prognostic genes, the expression heatmap of the differentiation timeline showed that *BBC3* gradually increased in expression during the third and terminal stages of differentiation, while *TCF7* exhibited a trend of initial increase followed by decline (**Figures 6A, B**). Furthermore, T*CF7* and *BBC3* were notably expressed in B cells, whereas other prognostic genes showed weaker expression in B cells (**Figure 6C**). Metabolic analysis revealed that IgE-MemB/PB enriched sterol and amino acid metabolism, suggesting this subpopulation may favor secretory lineages (e.g., ER/membrane lipid biogenesis and antibody glycosylation substrate supply). The GC-LZ-like subpopulation enriched for BCAA, alanine, aspartate, glutamate, and sulfur metabolism, indicating that the light zone selection period relies on amino acid carbon-nitrogen flux and glutathione antioxidant functions to maintain signaling and cell survival. The GC-DZ (Cycling) subpopulation enriched for pyrimidine, folate, and one-carbon metabolism aligns with the high demand for nucleotide synthesis and one-carbon donors during dark-phase proliferation. Conversely, the FolB IgM+CD23+ subpopulation enriched for glycerolipid metabolism and fatty acid chain elongation suggests this subset may participate in early-stage membrane lipid replenishment and receptor signaling platform remodeling (**Figure 6D**). The above findings provide an in-depth perspective that helps elucidate how B cell subsets function in immune responses through gene expression and metabolic pathways, while also revealing the potential clinical applications of these cellular characteristics.

### 3.9 RT-qPCR validation of prognostic genes

**Figure 7:**
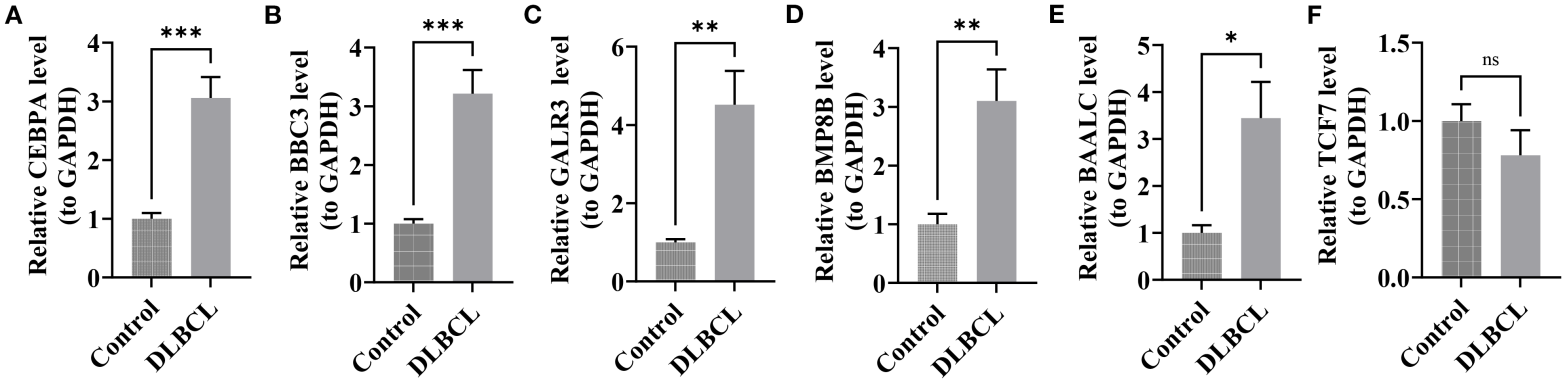
RT-qPCR validation of candidate genes in DLBCL. **(A)** *CEBPA* expression in DLBCL compared to controls. **(B)** *BBC3* expression in DLBCL compared to controls. **(C)** *GALR3* expression in DLBCL compared to controls. **(D)** *BMP88B* expression in DLBCL compared to controls. **(E)** *BAALC* expression in DLBCL compared to controls. **(F)** *TCF7* expression in DLBCL compared to controls. (all *P*< 0.05). “*” indicates P < 0.05, “**” indicates P < 0.01, and “***” indicates P < 0.001.

In DLBCL, the expression of *CEBPA*, *BBC3*, *GALR3*, *BMP8B*, and *BAALC* was markedly higher than in the control group (*P*< 0.05) (**Figures 7A–E**). Although *TCF7* expression tended to be downregulated in DLBCL, the differences were not significant, possibly due to the limited sample size (**Figure 7F**). The differential expression of these genes further underscored their prognostic value for DLBCL.

## 4 Discussion

DLBCL is a common, aggressive lymphoma with heterogeneous outcomes, presenting significant challenges in diagnosis and treatment (62). While mitochondrial dynamics (63) and mitophagy (64) are crucial for cellular homeostasis and are known to be involved in cancer progression, their specific role in DLBCL is not fully understood. In the present study, through integrative analysis of RNA-seq data and multimodal bioinformatics approaches, we systematically investigated the prognostic significance of MDRGs and MRGs in DLBCL.

Our study identified six pivotal prognostic biomarkers (*TCF7*, *CEBPA*, *BBC3*, *GALR3*, *BMP8B*, and *BAALC*) and established a robust risk score model demonstrating high predictive accuracy for patient outcomes. *TCF7*, a member of the TCF/LEF family, encodes the transcription factor *TCF-1*, which is a key modulator of the canonical Wnt/β-catenin signaling pathway (65). By binding to β-catenin, *TCF7* regulates gene expression and is essential for embryonic development, maintaining tissue homeostasis, stem cell preservation, and immune system regulation (66). Furthermore, *TCF1* (encoded by *TCF7*) may contribute to the maintenance of stemness and antitumor activity in CD8^+^ T cells by modulating mitochondrial function, particularly through the oxidative phosphorylation pathway (67). During HIV infection, downregulation of *TCF1* is associated with impaired mitochondrial function, which further compromises T-cell proliferative capacity and immune responses (68).Research has shown that *TCF7*-positive ancestral exhausted T cells in T-cell/histiocyte-rich large B-cell lymphoma can predict better responses to PD-1/PD-L1 blockade therapy (69). Our study results indicated that *TCF7* likely plays a crucial role in the pathogenesis and treatment response of DLBCL, potentially offering a novel therapeutic target or biomarker for this aggressive malignancy.

*CCAAT* enhancer binding protein alpha, known as *CEBPA*, a vital component of the *CEBP* family, encodes a key transcription factor widely involved in cell differentiation and metabolism regulation (70). Building upon prior evidence, it is proposed that *CEBPA* drives the transcriptional upregulation of circular RNA keratin 4(circKrt4), which then operates in the cytoplasm to disrupt mitochondrial integrity—specifically by interfering with the trafficking of mitochondria-anchored Glpk—suggesting a plausible mechanistic route through which circKrt4 may induce mitochondrial impairment (71). Mutations or abnormal expression of *CEBPA* are associated with various diseases, particularly in acute myeloid leukemia (AML) (72), where its mutation is considered a disease-driving factor. Studies have shown that overexpression of CAPG accelerates the malignancy of DLBCL cells, and CAPG expression is regulated by *CEBPA (* 73, 74). Although the direct role of *CEBPA* in DLBCL has not been extensively documented in the literature, our study found that *CEBPA* was significantly upregulated in DLBCL. It can be hypothesized that *CEBPA* could enhance the malignant growth of DLBCL cells by upregulating CAPG expression, thereby driving the progression of DLBCL.

*BBC3*, also known as *PUMA* (p53 upregulated modulator of apoptosis) (75), is a crucial member of the *BCL-2* family and encodes a pro-apoptotic protein (76). *BBC3* plays a key role in cellular responses to DNA damage and other stress signals, maintaining cellular and tissue homeostasis by promoting programmed cell death (77). Emerging evidence indicates that *BBC3* modulates mitochondrial function and triggers mitochondrial-mediated apoptosis, playing a pivotal role in mitochondrial trafficking and accumulation. Chaperone-mediated autophagy (CMA) influences *BBC3*’s intracellular transport—through regulating its degradation or stabilization—thereby critically modulating cell survival and death (76). Moreover, miR-222-3p may attenuate mitochondrial-mediated cell death following spinal cord injury by targeting both *BBC3* and Bim (also known as Bcl2l11), potentially contributing to improved neurological outcomes (78). Research has demonstrated that *BBC3* is significantly upregulated in DLBCL, suggesting that it promotes apoptosis in tumor cells, thereby inhibiting tumor growth and development. Since evading apoptosis is a crucial characteristic of tumor cells, the upregulation of *BBC3* may counteract this tendency, making tumor cells more susceptible to apoptotic signals and leading to cell death.

*GALR3*, a vital member of the galanin receptor family, is a G protein-coupled receptor (79). *GALR3* is widely expressed in the nervous system and regulates neurotransmission, mood balance, pain perception, and appetite control (80). By binding to the neuropeptide galanin, *GALR3* activates downstream pathways, including inhibiting cAMP production and regulating calcium ion channels, affecting neuronal excitability (81). It is thus speculated that the upregulation of *GALR3* in DLBCL may indirectly affect immune cell functions and immune factor secretion through the nervous system, alter the tumor microenvironment, and influence the disease progression of DLBCL.

*BMP8B*, belonging to the bone morphogenetic protein family, regulates embryonic development, bone formation, energy metabolism, and body temperature (82, 83). Recent studies have suggested its potential role in enhancing the invasiveness of DLBCL and its connection to stem cell-like properties (84). Moreover, studies in mice have revealed that mitochondrial dysfunction, indicated by alterations in markers such as peroxisome proliferator-activated receptor gamma coactivator 1-alpha(*Ppargc1a*) and PTEN induced putative kinase 1(*Pink1*), contributes to the downregulation of thermogenic markers including bone morphogenetic protein 8b (*Bmp8b*) and uncoupling protein 1(*Ucp1*), thereby promoting the whitening of inguinal brown adipose tissue and metabolic dysregulation (85). It has been reported that *BAALC* can bind to MAP3K1 and KLF4, participating in multiple cell signaling pathways (86). In cancers like AML, *BAALC* expression levels are correlated with prognosis, with high expression indicating poor prognosis and lower patient survival (87). These findings establish *BAALC* as a significant biomarker and potential therapeutic target in tumor research.

The constructed six-gene risk model (*TCF7*, *CEBPA*, *BBC3*, *GALR3*, *BMP8B*, and *BAALC*) demonstrated generalizability in an independent cohort with an AUC > 0.7, outperforming traditional prognostic indicators such as the IPI score and achieving comparable performance to recently developed gene models (e.g., DLBCL90) (88). Notably, this study is the first to focus on the mitochondrial function axis in metabolic regulation. Furthermore, a nomogram integrating clinical parameters (age, lactate dehydrogenase (LDH, ECOG performance status (PS), etc.) enhanced predictive accuracy (AUC > 0.7), providing a practical tool for individualized prognostic assessment.

Our research found that multiple pathways related to energy metabolism were significantly enriched, indicating disruption of cellular energy homeostasis in DLBCL development. Among the enriched pathways, the abnormal expression of MRGs in DLBCL could impact lysosome pathway activity, affecting cell metabolism and survival, aligning with earlier findings (89). Besides, abnormalities in the ribosome pathway might impact protein synthesis and cell proliferation (90), while spliceosome pathway abnormalities can lead to dysregulated gene expression (91). Collectively, these pathway abnormalities may drive DLBCL progression and drug resistance. The identified prognostic genes might regulate these pathways, influencing DLBCL biology and offering new diagnostic and therapeutic targets.

The CIBERSORT algorithm was employed to assess the relative amounts of various immune cells in our samples. Our study on immune infiltration showed notable variations in the types and amounts of immune cells among different risk categories and stages, indicating a potential role for immune dysregulation in DLBCL. Immune cells, particularly M0 macrophages and memory B cells, were found to be involved in the progression of DLBCL through mechanisms such as cytokine release, allograft rejection, apical junction, and coagulation (92). Indeed, understanding these relationships can help develop new immunotherapies and personalized treatment plans for DLBCL.

In the present study, B cells were identified as pivotal cellular components due to their significant differential expression of multiple prognostic genes across distinct subgroups. DLBCL originates from rapidly proliferating malignant B cells that originate from either germinal center or post-germinal center B cells. These neoplastic B cells undergo malignant transformation under various pathogenic factors, forming the cellular basis of DLBCL pathogenesis (93). Notably, constitutive activation of the B-cell receptor (BCR) signaling pathway plays a critical role in maintaining B-cell activation and survival. In DLBCL, somatic mutations in genes such as *CD79A*, *CD79B*, and *CARD11* drive ligand-independent persistent activation of BCR signaling, thereby providing sustained proliferative and survival signals that promote tumor progression (94). Furthermore, malignant B cells in lymphoid cancers leverage several methods to escape immune detection, such as decreasing the expression of major histocompatibility complex (MHC) molecules to evade detection by cytotoxic T cells, and overexpression of immune checkpoint molecules like PD-L1 to cause T cell exhaustion and weaken the anti-tumor immune response, ultimately facilitating tumor survival and expansion (95).

Based on these observations, we hypothesize that prognostic genes may exert their regulatory effects through multifaceted mechanisms involving B cell biology in DLBCL pathogenesis. The comprehensive roles of B cells span from initial malignant transformation to subsequent tumor maintenance and immune escape processes. The tumor microenvironment demonstrated a strong association between prognostic genes and both M0 macrophages and memory B cells. The prognostic genes might affect the function and interactions of these key cell types, driving DLBCL progression. For instance, trajectory analysis revealed that *CEBPA*, *BAALC*, and *BMP8* are expressed during the middle stage of B cell development, whereas *TCF7* and *BBC3* are highly expressed during the early and late stages, suggesting that the influence of prognostic genes is dependent on the specific B cell differentiation stage.

However, this study has limitations that should be acknowledged. First, the reliance on publicly available databases introduces a risk of bias from cohort heterogeneity (e.g., treatments, geography), requiring multi-center validation for generalizability. Second, the precise mechanisms linking prognostic genes to mitochondrial dynamics/autophagy remain to be fully elucidated. Future experiments, such as knocking down or overexpressing these genes *in vitro* and *in vivo* to further explore their biological roles. Third, the drug sensitivity analyses excluded emerging therapies like CAR-T and mitochondrial-targeting agents (e.g., IACS-010759), necessitating expanded pharmacogenomic data. Finally, the prognostic model’s clinical utility and predictive power warrant further validation with robust clinical evidence. To further validate the prognostic usefulness of our model, future studies that are large-scale, multi-center, and prospective are crucial. These validation experiments constitute the critical next phase of our research and will be the primary focus of our subsequent work.

## 5 Conclusion

In the present study, six prognostic genes related to mitochondrial dynamics and mitophagy in DLBCL (*TCF7*, *CEBPA*, *BBC3*, *GALR3*, *BMP8B*, and *BAALC*). A robust risk model was developed from these genes, demonstrating strong and stable predictive ability for DLBCL prognosis. The findings reveal a critical association between high-risk disease and specific alterations in the tumor microenvironment—particularly involving M0 macrophages—coupled with epitranscriptomic dysregulation mediated by m6A modifications. This insights offers a novel theoretical framework and approach for the early diagnosis and advancing targeted treatment of DLBCL, as well as for exploring mitochondrial dynamics and mitophagy mechanisms.

## Data Availability

The datasets presented in this study can be found in online repositories. The names of the repository/repositories and accession number(s) can be found in the article/**Supplementary Material**.
