# Supplementary material for: Prognostic genes related to mitochondrial dynamics and mitophagy in diffuse large B-cell lymphoma are identified and validated using an integrated analysis of bulk and single-cell RNA sequencing
Source: Front Immunol. 2025 Oct 9;16:1686948. doi: 10.3389/fimmu.2025.1686948 (PMC12546034; doi:10.3389/fimmu.2025.1686948)
Supplement: Supplementary file 1 [file DataSheet1.docx]

**
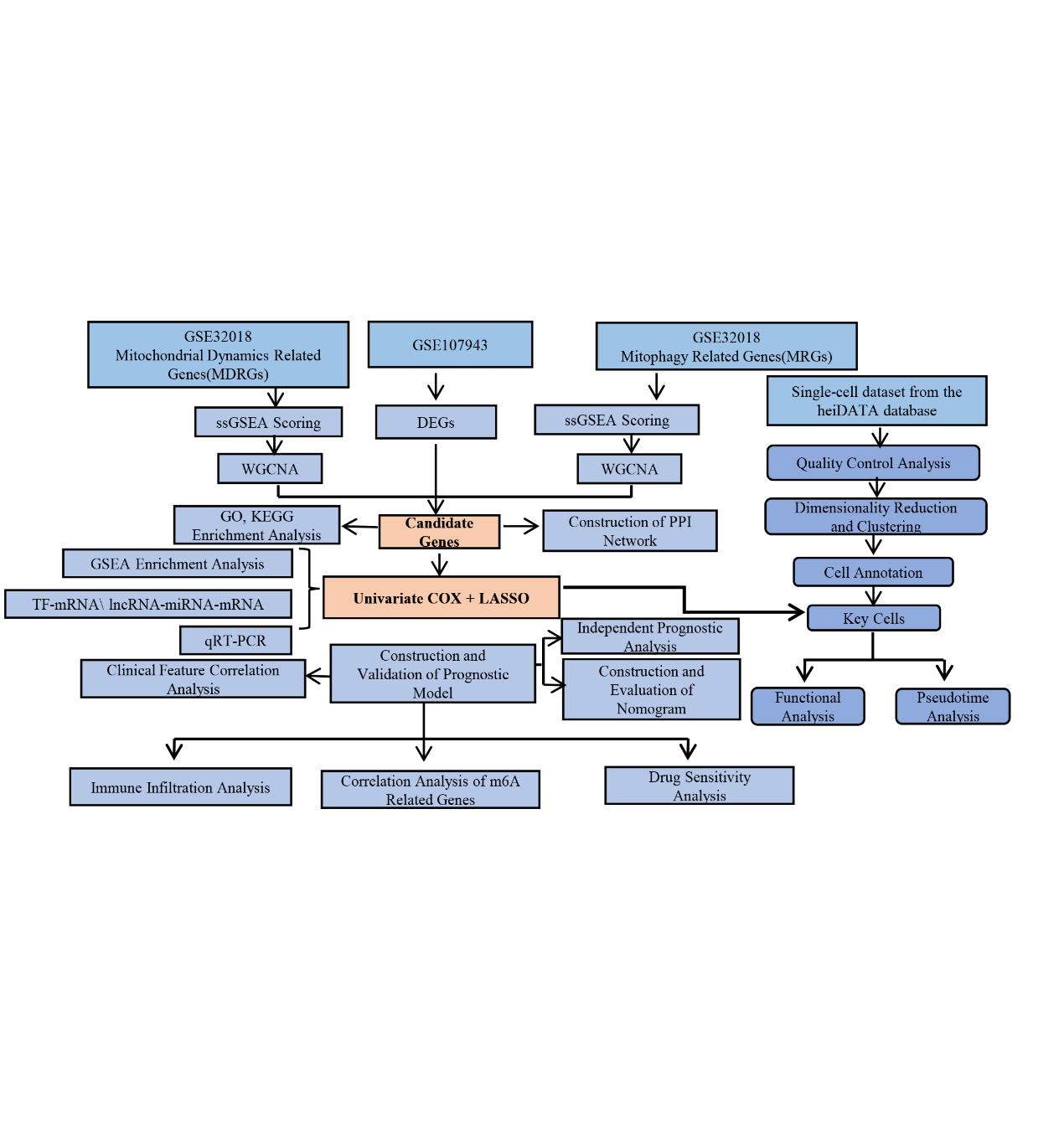
Fig. S1 Flow diagram of the trial.**

**
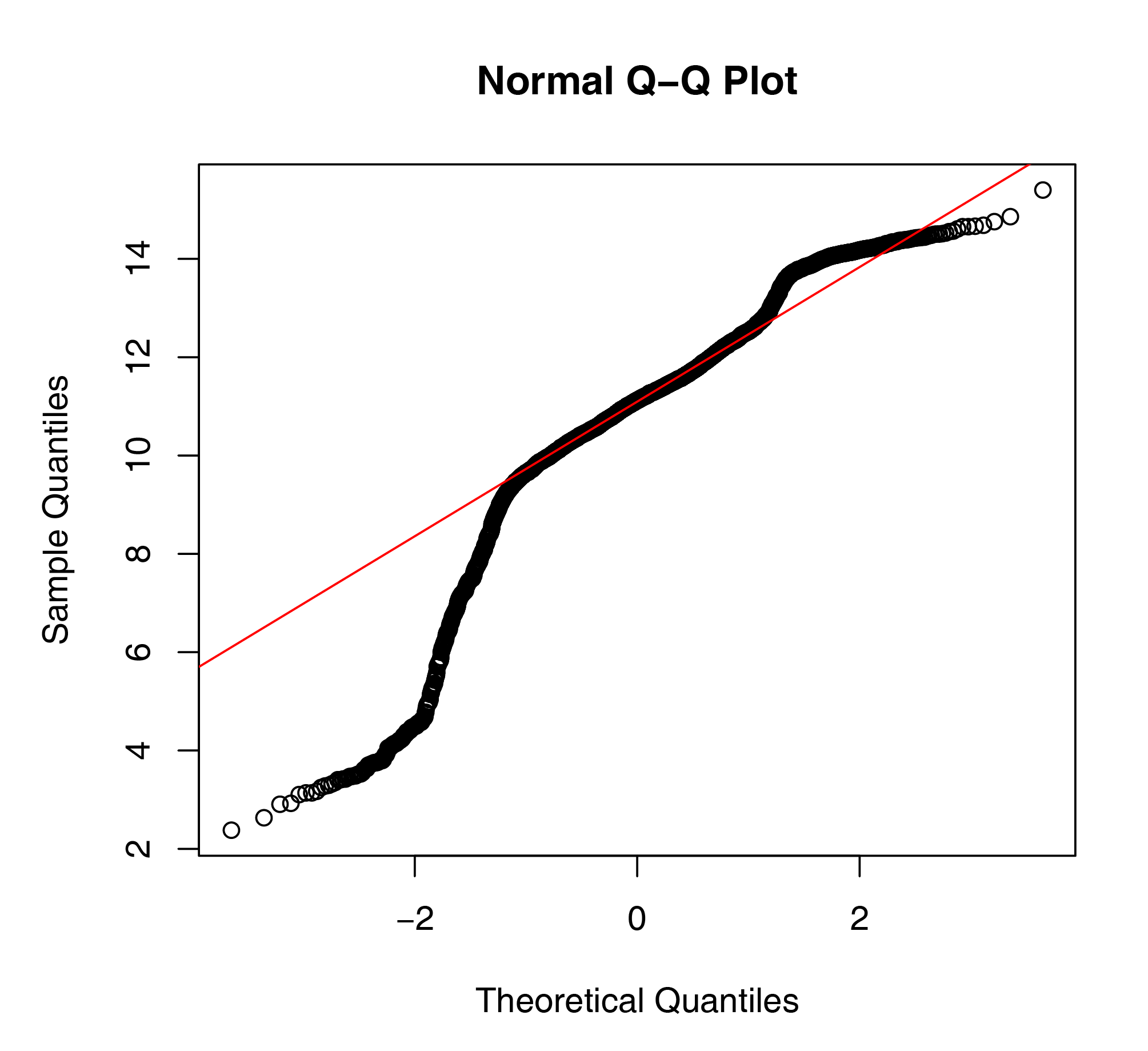
**

**Fig. S2 QQ plot for assessing whether data follows a normal distribution.**

**
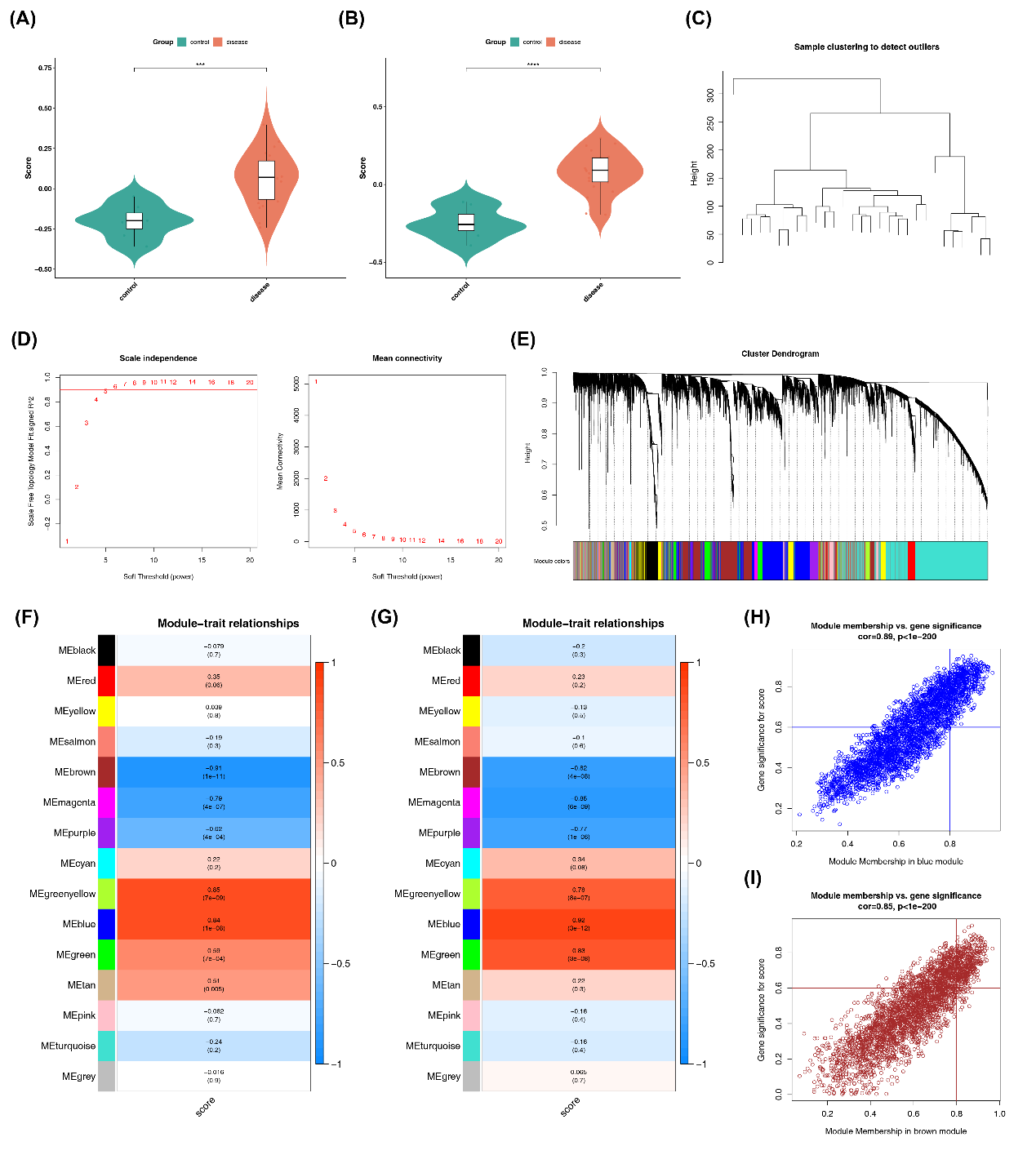
 Fig. S3 Analysis of MDRGs/MRGs in DLBCL progression.** (A-B) Elevated scores of MDRGs and MRGs in different groups within GSE32018. (C) Hierarchical clustering results of outlier detection (cutHeight = 290). (D) Scale-free network display of the best soft threshold from WGCNA. (E) Gene co-expression network with 14 clustered modules (the grey module excluded due to poor clustering). (F-G) Key module-trait correlations: The blue module represents strong positive correlations with MDRGs (r = 0.84) and MRGs (r = 0.92), and the brown module represents strong negative correlations with MDRGs (r = -0.91) and MRGs (r = -0.82). (H-I) Final selection of 931 high-confidence key module genes (|MM| > 0.8, |GS| > 0.6).

**
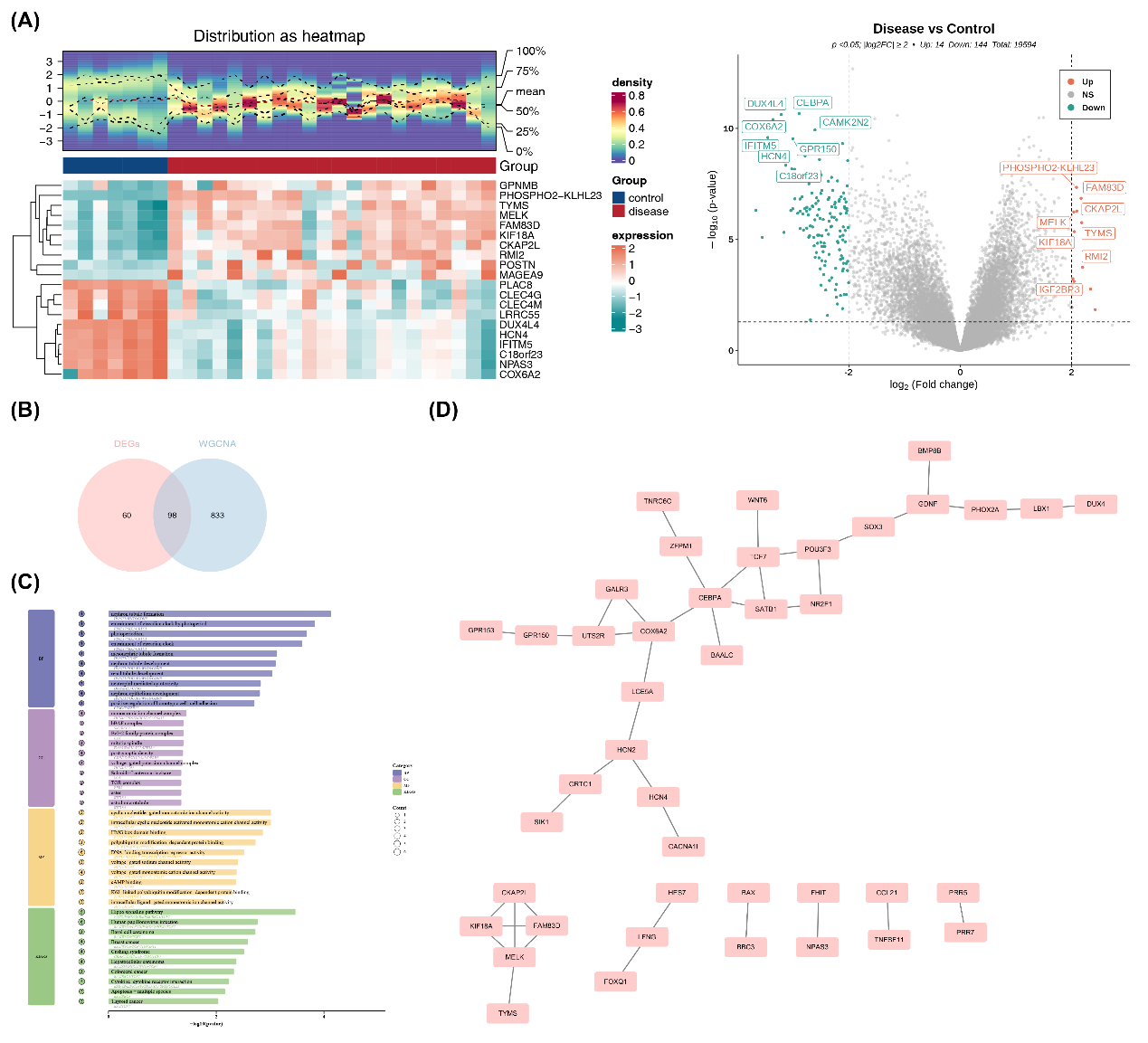
 Fig. S4 Functional and interaction analysis of DLBCL-associated genes.** (A) DEGs analysis in dataset GSE32018. The red and green dots represent the upregulated and downregulated genes, respectively, with criteria of *P*-value < 0.05 and |log2 fold change|> 2. (B) The intersection of DEGs and key module genes yielded 98 candidate genes. (C) Significant enrichment of candidate genes in GO terms and KEGG pathways (*P* < 0.05). (D) PPI network comprising 42 proteins and 41 interaction pairs, highlighting complex protein-level regulation of candidate genes.


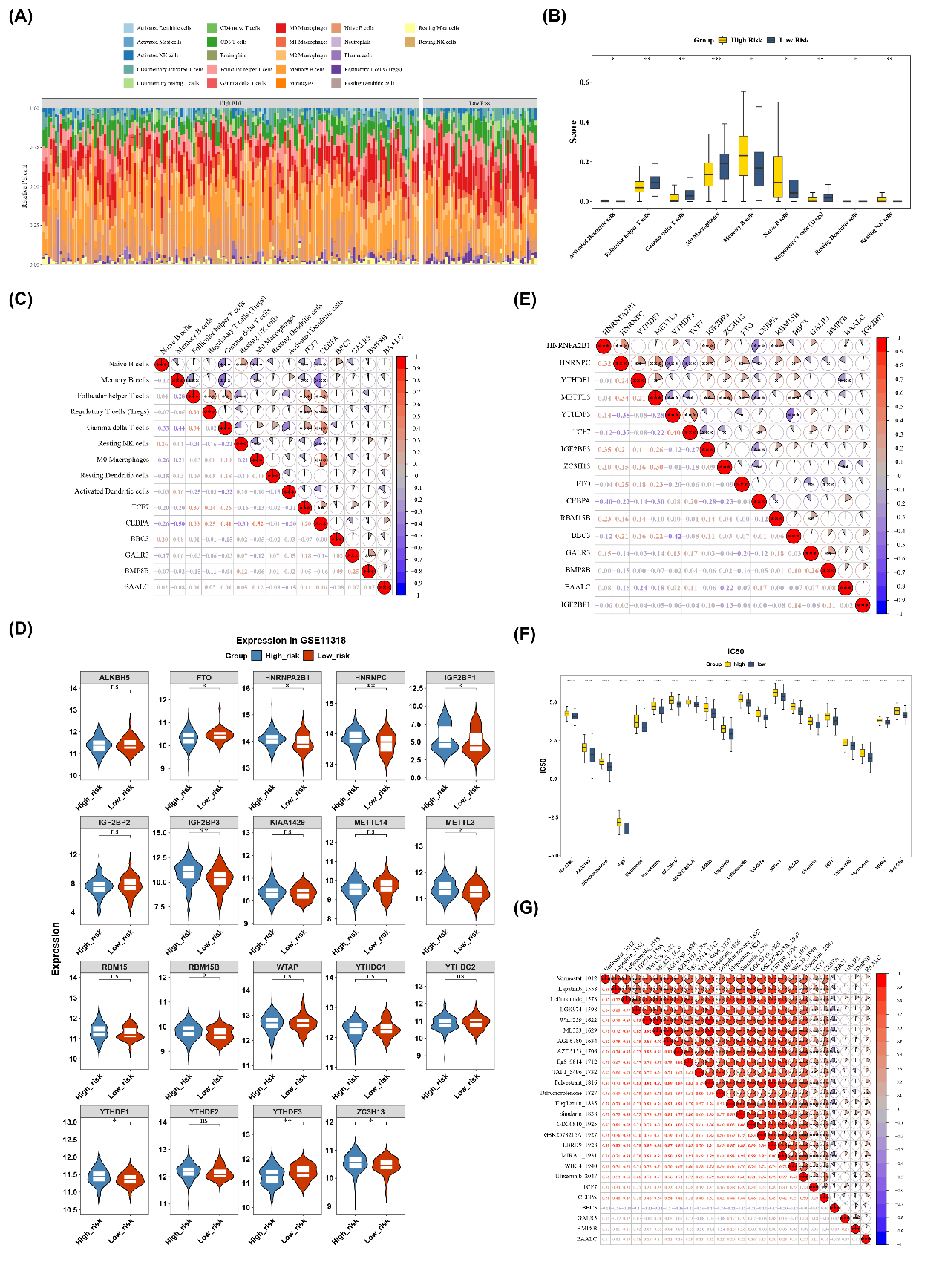


**Fig. S5 Tumor microenvironment (TME), m6A modification, and drug sensitivity analysis in DLBCL risk groups.** (A) TME profiles of high-risk (HRG) versus low-risk (LRG) groups. (B) Eight differentially infiltrated immune cell types. (C) Correlations between prognostic genes and immune cells. (D) Differential expression of m6A-related genes between HRG and LRG. (E) Associations between prognostic genes and m6A regulators. (F) Drug sensitivity differences between HRG and LRG showed lower IC50 values for dihydrorotenone, elephantin, and fulvestrant in LRG (*P* < 0.0001). (G) Correlations between prognostic genes and therapeutic drug sensitivity.


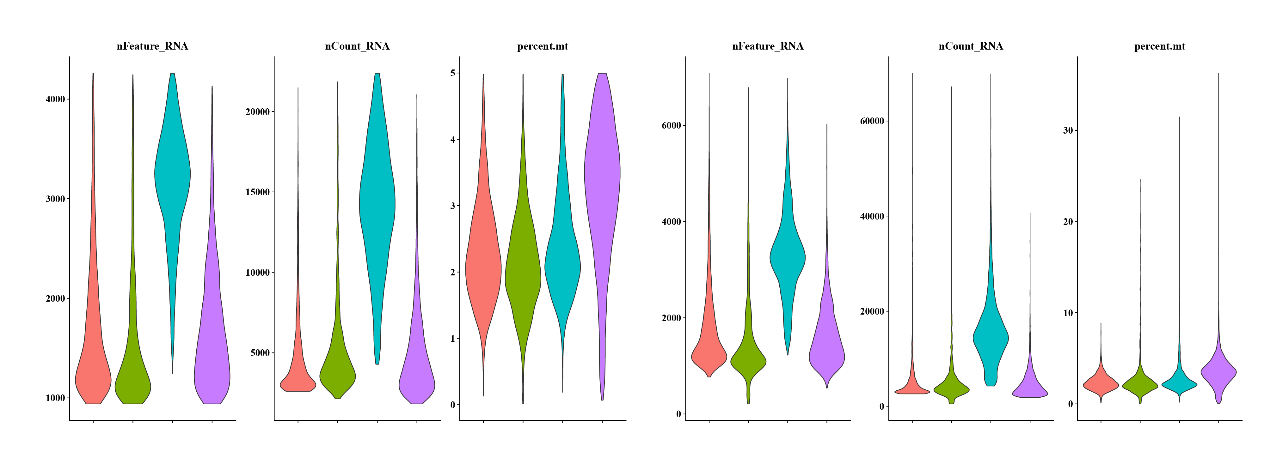


**Fig. S6 Quality control metrics of single-cell RNA sequencing data after and before filtering.** (A) The first three violin plots of the same metrics after applying quality filters. The applied thresholds (e.g., minimum gene count, maximum mitochondrial percentage) successfully removed low-quality cells, resulting in a uniform, high-quality dataset suitable for downstream analyses such as clustering and differential expression. (B) The last three violin plots displaying the distribution of three key QC metrics across all cells in the raw, unfiltered dataset. The wide distributions and extreme values (e.g., a large population of cells with very low gene/UMI counts indicative of empty droplets, and cells with very high mitochondrial content indicative of apoptosis or necrosis) highlight the necessity for rigorous quality filtering.

Metrics shown: nFeature_RNA (number of genes detected per cell), nCount_RNA (total number of UMIs detected per cell), and percent.mt (percentage of mitochondrial gene counts).


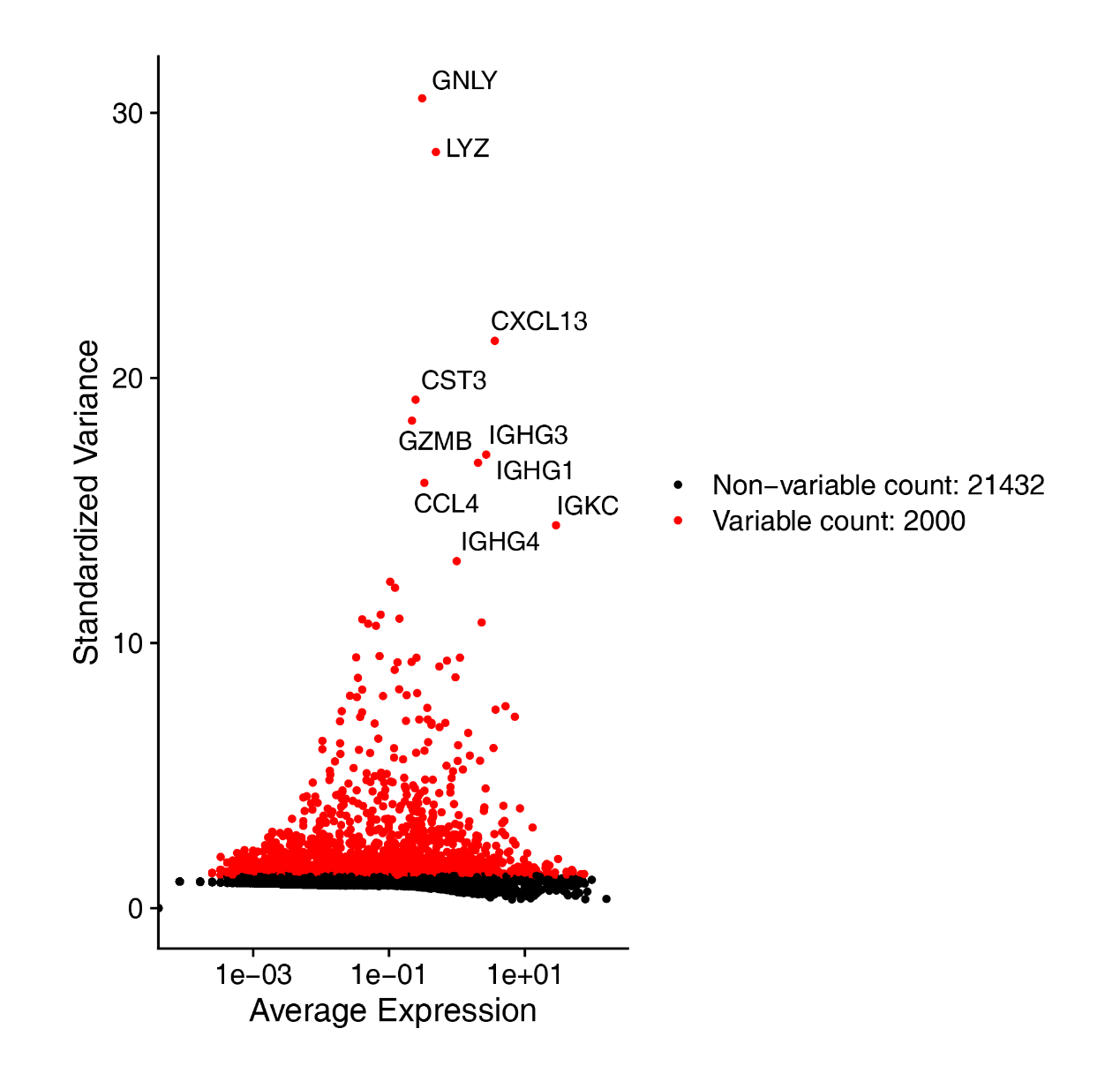


**Fig. S7. Identification of highly variable genes for downstream dimensionality reduction.** A scatter plot of gene variance (dispersion) against average expression across all cells. A total of 2,000 genes (red) were identified as highly variable features, which exhibit high cell-to-cell variation in expression levels and are likely to drive biological heterogeneity. The remaining 21,432 genes (grey) showed low variation. Several known cell marker genes are labeled among the highly variable genes, validating the selection process. These variable genes were used as input for principal component analysis (PCA) and subsequent clustering.


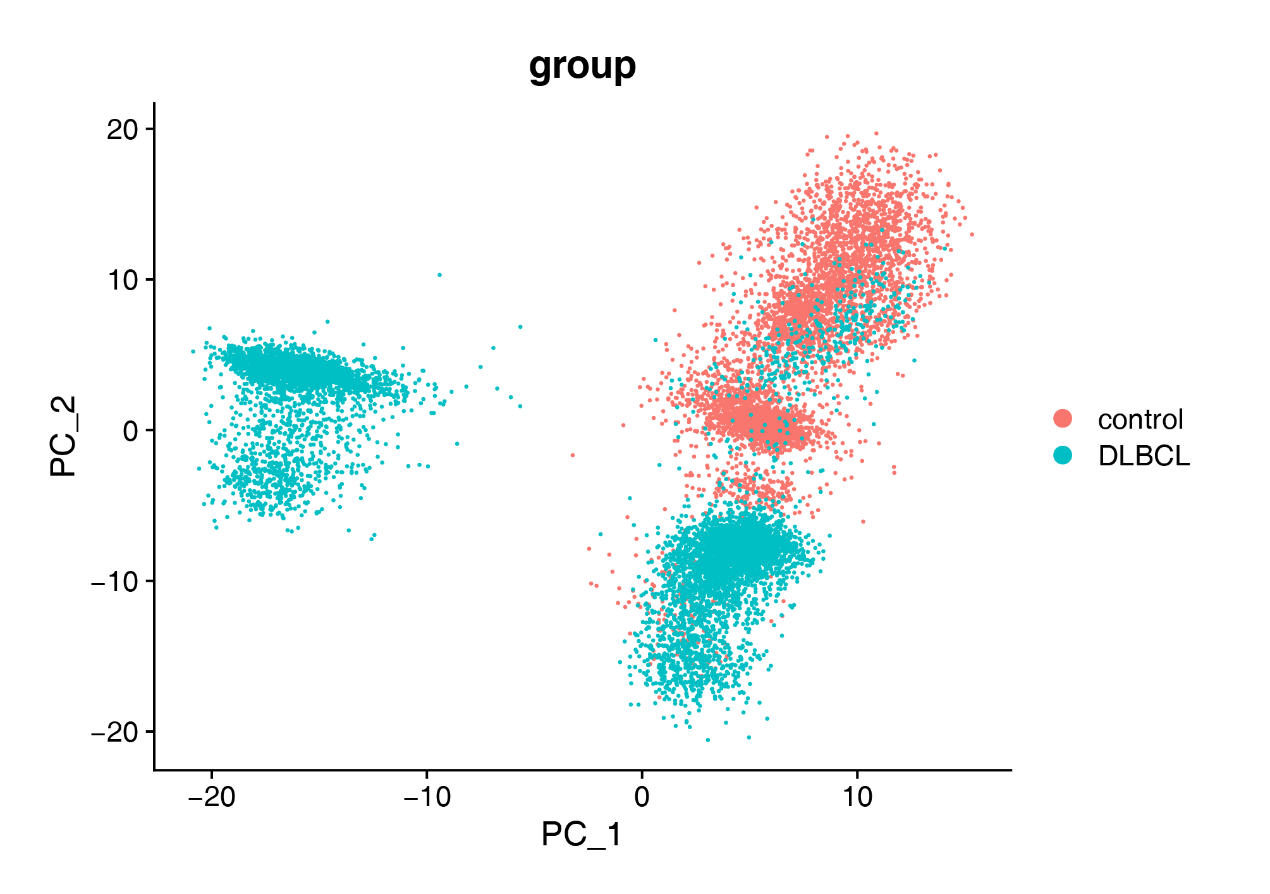


**Fig. S8. Principal component analysis (PCA) reveals distinct transcriptional profiles between control and DLBCL groups.** Scatter plot of the first two principal components computed from the highly variable genes. Each point represents a single cell (or sample), colored by its group assignment. The clear separation of control and DLBCL cells primarily along the first principal component indicates substantial transcriptomic differences driven by the disease state. PCA was performed following quality control and variable feature selection.


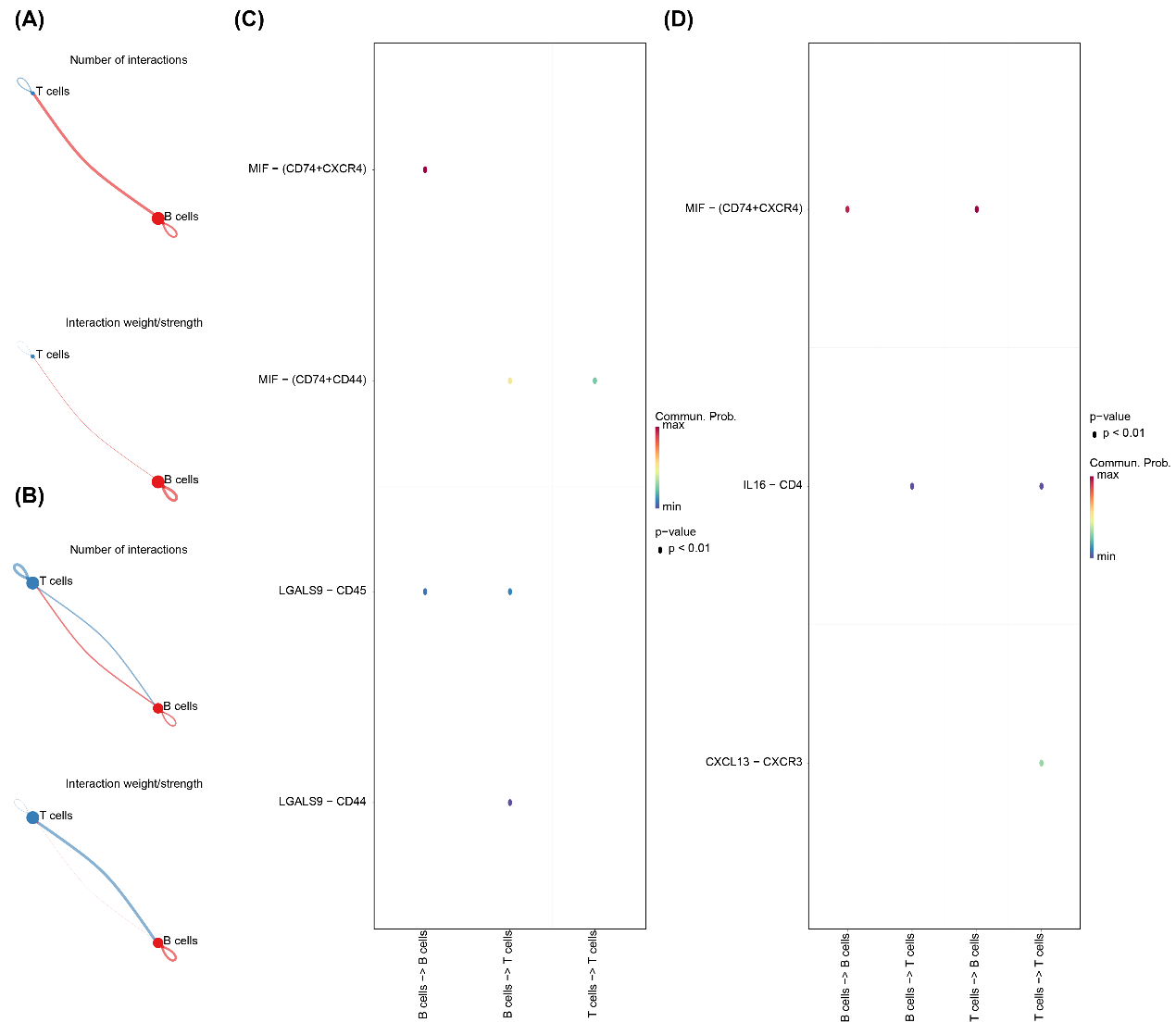


**Fig. S9 Results of cell communication.** (A) Cell communication analysis of the disease group. (B) Cell communication analysis of the control group. (C) Cell communication bubble chart of the disease group. (D) Cell communication bubble chart of the control group.


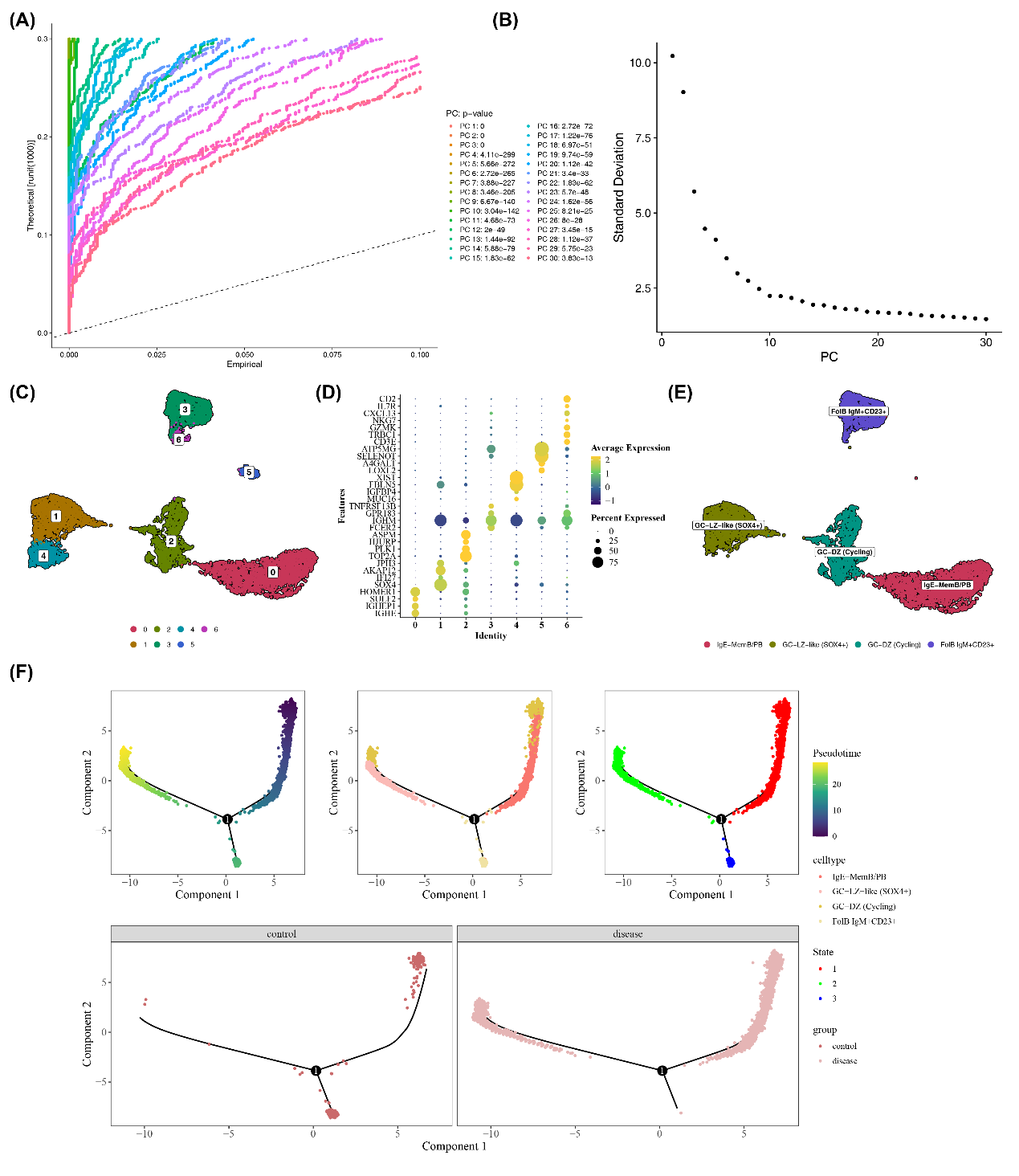


**Fig. S10 Comprehensive analysis of key cell subpopulations.** (A-B) PCA analysis results, showing the distribution of different samples across principal components. (C) UMAP plot of key cell subpopulations, revealing clustering patterns in a low-dimensional space. (D) Heatmap of marker gene expression for different subpopulations, used to annotate cell subpopulations. (E) Annotated UMAP plot of key cell subpopulations, further illustrating the distribution of cell types in UMAP space. (F) Visualization of pseudo-time analysis results for key cell subpopulations, demonstrating dynamic changes during differentiation.

**Table S1.** **List of 23 mitochondrial dynamics-related genes (MDRGs) curated from the literature.**

| Gene_symbol |
| --- |
| ARMC10 |
| DNM1L |
| FIS1 |
| MFF |
| MIEF1 |
| MIEF2 |
| MTFP1 |
| MTFR1 |
| MTFR2 |
| MUL1 |
| OMA1 |
| RAB24 |
| SLC25A46 |
| SPIRE1 |
| STX17 |
| ARL2 |
| MFN1 |
| MFN2 |
| MIGA1 |
| MIGA2 |
| MTCH2 |
| OPA1 |
| PLD6 |

**Table S2. List of 29 mitophagy-related genes (MRGs) obtained from the Reactome database.**

| Gene_symbol |
| --- |
| ATG12 |
| ATG5 |
| CSNK2A1 |
| CSNK2A2 |
| CSNK2B |
| FUNDC1 |
| MAP1LC3A |
| MAP1LC3B |
| MFN1 |
| MFN2 |
| MTERF3 |
| PGAM5 |
| PINK1 |
| PRKN |
| RPS27A |
| SQSTM1 |
| SRC |
| TOMM20 |
| TOMM22 |
| TOMM40 |
| TOMM5 |
| TOMM6 |
| TOMM7 |
| TOMM70 |
| UBA52 |
| UBB |
| UBC |
| ULK1 |
| VDAC1 |

**Table S3. Primer sequences used for quantitative real-time PCR (qRT-PCR) analysis.**

| **Primers** | **Sequences (5’ to 3’)** | |
| --- | --- | --- |
| TCF7-F | CCAAGAATCCACCACAGGAGG | |
| TCF7-R | TGCTGTACCTGTGTGCTCTG | |
| CEBPA-F | ATTCCGGTGCCTCCTGAAAG |  |
| CEBPA-R | GAAGGAGGCAGGAAACCTCC |  |
| BBC3-F | GATTTGTGGTCCTCAGCCCT |  |
| BBC3-R | CTCGTACTGTGCGTTGAGGT |  |
| GALR3-F | TGGCTGACCTCTGCTTCATC |  |
| GALR3-R | AGACAGCAGCCAGCGTAAAG |  |
| BMP8B-F | CCTGCATGAATGCCACCAAC |  |
| BMP8B-R | TTCCTCTCACACCAAGGCAT |  |
| BAALC-F | CCTGCACTCGGGCTAAAAGA |  |
| BAALC-R | GCCGTGAAGGACACTGAAGA |  |
| GAPDH-F | ATGGGCAGCCGTTAGGAAAG |  |
| GAPDH-R | AGGAAAAGCATCACCCGGAG |  |

**Table S4. Summary of candidate prognostic genes in DLBCL.**

| **Gene** | ***P*** |
| --- | --- |
| *TYMS* | 0.384280662 |
| *TCF7* | 0.137791517 |
| *CEBPA* | 0.075444741 |
| *SATB1* | 0.10912524 |
| *GPR153* | 0.103270174 |
| *BBC3* | 0.325741244 |
| *TNRC6C* | 0.086243568 |
| *GALR3* | 0.490221302 |
| *BMP8B* | 0.385842975 |
| *PRR7* | 0.626232846 |
| *BAALC* | 0.669558692 |
| *NPAS3* | 0.306977037 |

**Table S5. The clinical factors incorporated in the DLBCL Prognostic Model.**

| **Factor** | ***P*** |
| --- | --- |
| Risk score | 0.863561529 |
| Age | 0.843681361 |
| LDH ratio | 0.27964976 |
| Extranodal | 0.091474681 |
| ECOG PS | 0.062801896 |
| Stage | 0.060305061 |

**Table S6. Marker genes used for cell type annotation.**

| **Cell type** | **Cluster** | **Marker genes** |
| --- | --- | --- |
| IgE−MemB/PB | 0 | "IGHE", "IGHEP1", "SULF2", "HOMER1" |
| GC−LZ−like (SOX4+) | 1 | "SOX4", "IFI27", "AKAP12", "JPH3" |
| GC−DZ (Cycling) | 2 | "TOP2A", "PLK1", "HJURP", "ASPM" |
| FolB IgM+CD23+ | 3 | "FCER2", "IGHM", "GPR183", "TNFRSF13B" |
| Non−B (Epithelial−like) | 4 | "MUC16", "IGFBP4", "FBLN5", "XIST" |
| Non−B (Stromal/Metab.) | 5 | "LOXL2", "A4GALT", "SELENOT", "ATP5MG" |
| T | 6 | "CD3E", "TRBC1", "GZMK", "NKG7", "CXCL13", "IL7R", "CD2" |
